# Lithiation Mechanism in High-Entropy Oxides as Anode Materials for Li-Ion Batteries: An Operando XAS Study

**DOI:** 10.1021/acsami.0c13161

**Published:** 2020-10-30

**Authors:** P. Ghigna, L. Airoldi, M. Fracchia, D. Callegari, U. Anselmi-Tamburini, P. D’Angelo, N. Pianta, R. Ruffo, G. Cibin, Danilo Oliveira de Souza, E. Quartarone

**Affiliations:** † Department of Chemistry, University of Pavia, Via Taramelli 16, 27100 Pavia, Italy; ‡ Department of Chemistry, University of Rome La Sapienza, P.le A. Moro 5, 00185 Rome, Italy; § Department of Materials Science, University of Milano Bicocca, Via Cozzi 55, 20156 Milano, Italy; ∥ Diamond Light Source Ltd., Harwell Science and Innovation Campus, OX11 0DE Didcot, U.K.; ⊥ Elettra-Sincrotrone Trieste, s.s. 14 km 163,500 in Area Science Park, 34149 Basovizza, TS, Italy

**Keywords:** high-entropy oxides, anodes, lithium-ion batteries, operando XAS, lithiation mechanism

## Abstract

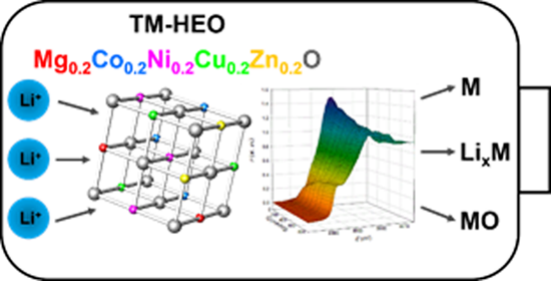

High-entropy oxides based on transition metals, such as Mg_0.2_Co_0.2_Ni_0.2_Cu_0.2_Zn_0.2_O (TM-HEO), have recently drawn special attention as potential anodes in lithium-ion batteries due to high specific capacity and cycling reversibility. However, the lithiation/delithiation mechanism of such systems is still controversial and not clearly addressed. Here, we report on an operando XAS investigation into TM-HEO-based anodes for lithium-ion cells during the first lithiation/delithiation cycle. This material showed a high specific capacity exceeding 600 mAh g^–1^ at 0.1 C and Coulombic efficiency very close to unity. The combination of functional and advanced spectroscopic studies revealed complex charging mechanisms, developing through the reduction of transition-metal (TM) cations, which triggers the conversion reaction below 1.0 V. The conversion is irreversible and incomplete, leading to the final collapse of the HEO rock-salt structure. Other redox processes are therefore discussed and called to account for the observed cycling behavior of the TM-HEO-based anode. Despite the irreversible phenomena, the HEO cubic structure remains intact for ∼60% of lithiation capacity, so proving the beneficial role of the configuration entropy in enhancing the stability of the HEO rock-salt structure during the redox phenomena.

## 1 Introduction

Lithium metal is an ideal anode for the next-generation Li-ion batteries (LIB) due to the highest theoretical capacity and lowest electrochemical potential among all of the possible candidate materials.^1^ Unfortunately, the safety risk is still a critical issue for practical applications, despite huge progress has been recently made to stabilize the Li-metal anode, thanks to new chemical strategies, advanced investigation tools, and nanotechnology approaches.^1,2^ The request for active systems as anode alternatives to Li is, therefore, strongly compelling.

The first commercial choice was graphite. However, its low theoretical capacity (372 mAh g^–1^) and unsafe charging profile at high current densities promoted the search for alternative negative electrodes. Different innovative materials were consequently proposed in the literature with better electrochemical properties in terms of both potential and capacity, such as graphene,^3^ silicon-based systems,^4,5^ including Si/C composites,^6^ and a huge family of metal oxides and oxysalts,^7^ typically nanostructured.^8^

As known, research on advanced anodes for LIBs is focused on materials that are electrochemically active through three different mechanisms:^7^ (i) intercalation–deintercalation mechanism based on transition-metal (TM) oxides and other compounds with a 2D layered or 3D network structure, where Li can reversibly intercalate without any crystal structure collapse; (ii) metals than can form alloys/intermetallic compounds with Li, whose alloying/dealloying reactions are responsible for the reversible capacity during Li cycling; (iii) conversion mechanism, applicable to TM oxides, fluorides, oxyfluorides, sulfides, etc., which react with Li to give the corresponding reduced metals and Li_2_O. Lithium oxide can easily decompose to form metal and oxygen only if the TM oxide is nanosized, thus giving rise to Li cycling and large and reversible capacity at suitable potentials.

Very recently, an emerging class of materials is catching on based on the concept of multiple principal elements in equimolar or near-equimolar ratios, whose design can stabilize a single-phase structure of solid solutions by means of rigorous control of the configurational entropy (*S*_config_).

These kinds of materials, known as high-entropy materials (HEMs), may be properly designed with tailorable properties (for instance, mechanical, thermal, magnetic, dielectric), making them potentially suitable for a wide spectrum of technologies.^9^

The high-entropy concept has been first applied to nanostructured alloys^9^ and, more recently, also to other systems, like oxides, where up to five cations can be introduced to occupy the same Wyckoff position of the same crystal structure, thus increasing *S*_config_.^10^ The pioneering system was an equimolar solid solution of MgO, CoO, NiO, CuO, and ZnO, yielding the Mg_0.2_Co_0.2_Ni_0.2_Cu_0.2_Zn_0.2_O compound with the rock-salt structure,^10^ labeled in the following as TM-HEO, transition-metal high-entropy oxide.

It was recently shown that not only cation but also anion stoichiometry could be modulated to preserve the rock-salt structure of TM-HEO. Multianionic and multicationic high-entropy oxyhalides were synthesized by introducing an additional halide (X = F, Cl) into the HEO rock-salt structure, where only oxygen ions occupy the anion site, without any drastic strain. The presence of F^–^ or Cl^–^ must be charge-compensated by a monovalent cation M^+^, as Li^+^ or Na^+^, to give M*_x_*(Co_0.2_Cu_0.2_Mg_0.2_Ni_0.2_Zn_0.2_)OF*_x_*.^11^

Inspired by the well-known electrochemistry of the binary oxides MO (M = Mn, Fe, Co, Ni, Cu), TM-HEO was recently explored as a novel anode material for LIBs. In principle, it was found that entropy stabilization positively affects the capacity retention of the multicomponent oxide,^12,13^ leading to increased cycling stability than the individual MO.^14^ Indeed, high process reversibility and long-term cyclability over 900 cycles with a specific capacity higher than 650 mAh g^–1^ were observed in half-cells including conventional liquid electrolytes and microsized HEO as an anode. Such promising performances are evident only if all TMs are present in the structure. Conversely, significant cell failures are observed if one element is removed, especially Co.^14^

By considering that TM-HEO contains metal oxides in the rock-salt structure, it plausible to suppose that the mechanism involved during the Li storage and cyclability is the conversion reaction of some of the cations (e.g., Co^2+^, Ni^2+^, Cu^2+^): MO + 2Li^+^ + 2e^–^ → M + Li_2_O. The other cations, as Mg^2+^, acts as a kind of matrix, stabilizing the rock-salt phase and maintaining intact the structure during the redox process. However, the role of Mg^2+^ in preserving the HEO structure has been explained by considering Mg electrochemically inactive in the given potential window.^13^ On the other hand, it should be recalled that a large fraction of Li^+^ ions can be easily inserted mostly for two reasons: (i) defects, likely oxygen vacancies, and (ii) charge compensation in the system, achieved through the oxidation of some elements, for instance, Co^2+^ to Co^3+^.^15^

Despite these promising results, the reaction mechanism of TM-HEO as an anode for LIBs is not fully addressed, especially for what concerns the reversibility of the conversion process (decomposition of Li_2_O and metal oxidation to TM-HEO).

Based on XRD, TEM, and electron diffraction, Sarkar and co-workers recently proposed a lithiation mechanism that significantly differs from the conventional conversion reaction.^13^ In that study, it was shown that during the lithiation step, some cations (like Co^2+^ and Cu^2+^) are reduced to the corresponding metals. This is proved by the gradual disappearance of HEO XRD reflections. However, electron diffraction, performed on the cycled sample, reveals the presence of the rock-salt structure even in the fully lithiated phase, so confirming that other cations, such as Mg^2+^, stabilize the cubic structure, which is consequently preserved during the electrochemical reaction.

The metal nuclei, visible only by SAED due to their sizes smaller than the X-ray coherence length, grow inside the rock-salt structure, introducing defects that suppress the long-range order. The trapped metals can easily diffuse back during the oxidation step to restore the HEO structure after delithiation. However, the final XRD pattern is that of an amorphous system, with no HEO reflection evident after the first lithiation cycle. The presence of the rock-salt structure throughout the conversion mechanism is evident, in fact, only by SAED.^13^ However, this is not definitive proof that such a structure could be specifically referred to HEO rather than a single binary oxide such as MgO, CoO, or NiO, all of them having the same rock-salt crystal structure.

In summary, it was concluded that in the conversion reaction of TM-HEO, Mg^2+^ ions contribute to stabilize the phase, whereas the other cations, Co^2+^, Ni^2+^, Zn^2+^, and Cu^2+^, are responsible for the reversible capacity.^16^

Considering the promising functional properties of HEOs in LIBs, a deeper insight into the structural and electronic evolution of the cubic high-entropy oxide during the electrochemical process is fundamental to better clarify the reaction mechanism and to further optimize the system, in terms of structure and composition. A lack of translational order in the products of the conversion reaction asks for the application of a short-range probe. In this respect, X-ray absorption spectroscopy (XAS) is the selection tool, as it is sensitive to the local chemical environment of atoms in terms of both neighboring and electronic structures (oxidation state). In addition, hard X-rays have a quite large penetration depth in the matter, rendering in situ and operando experiments possible.

Herein, by applying ex situ and in operando XAS by X-ray absorption near-edge structure (XANES) at the Co, Ni, and Cu K-edges, we investigated the electronic and local structure evolution on the Mg_0.2_Co_0.2_Ni_0.2_Cu_0.2_Zn_0.2_O anode in LIBs to detail the reaction mechanism. Our results demonstrate, for the first time to our knowledge, that the lithiation in TM-HEO is irreversible and involves a reactive path where the TMs reduce in sequence to the metallic state: the residual reversible capacity is given by an alloying–dealloying mechanism involving Zn and Mg; we believe that such understanding can advance the development of high-performance HEOs electrodes for Li^+^- and Na^+^-ion batteries.

## 2 Experimental Section

### 2.1 Synthesis and Structural and Chemical Characterization of (Mg_0.2_Co_0.2_Ni_0.2_Cu_0.2_Zn_0.2_)O

(Mg_0.2_Co_0.2_Ni_0.2_Cu_0.2_Zn_0.2_)O (TM-HEO) was synthesized by a conventional solid-state reaction starting from the commercial metal oxides (MgO, ZnO, CuO, NiO, CoO; Aldrich). The starting materials were mixed in the proper stoichiometry and subsequently treated at 1000 °C for 6 h, after a temperature ramp of 10 °C min^–1^. Highly pure TM-HEO with ∼2–5 μm sized particles was produced, as evidenced by the SEM images of Figure S1a, whose rock-salt structure was confirmed by powder XRD (see Figure S1b). The EXAFS at the Co, Ni, Cu, and Zn K-edges, reported in Figure S1c, suggests that TM-HEO is also homogeneous concerning the chemical composition. The spectra show impressive similarities for all of the four cations (see below for experimental details) and appear extremely similar to those reported by Rost et al.^10^ Taking into account that EXAFS is sensitive to the radial distribution function around the photoabsorber, this result confirms that the local chemical environment of Co, Ni, Cu, and Zn is very similar.

**Figure 1:**
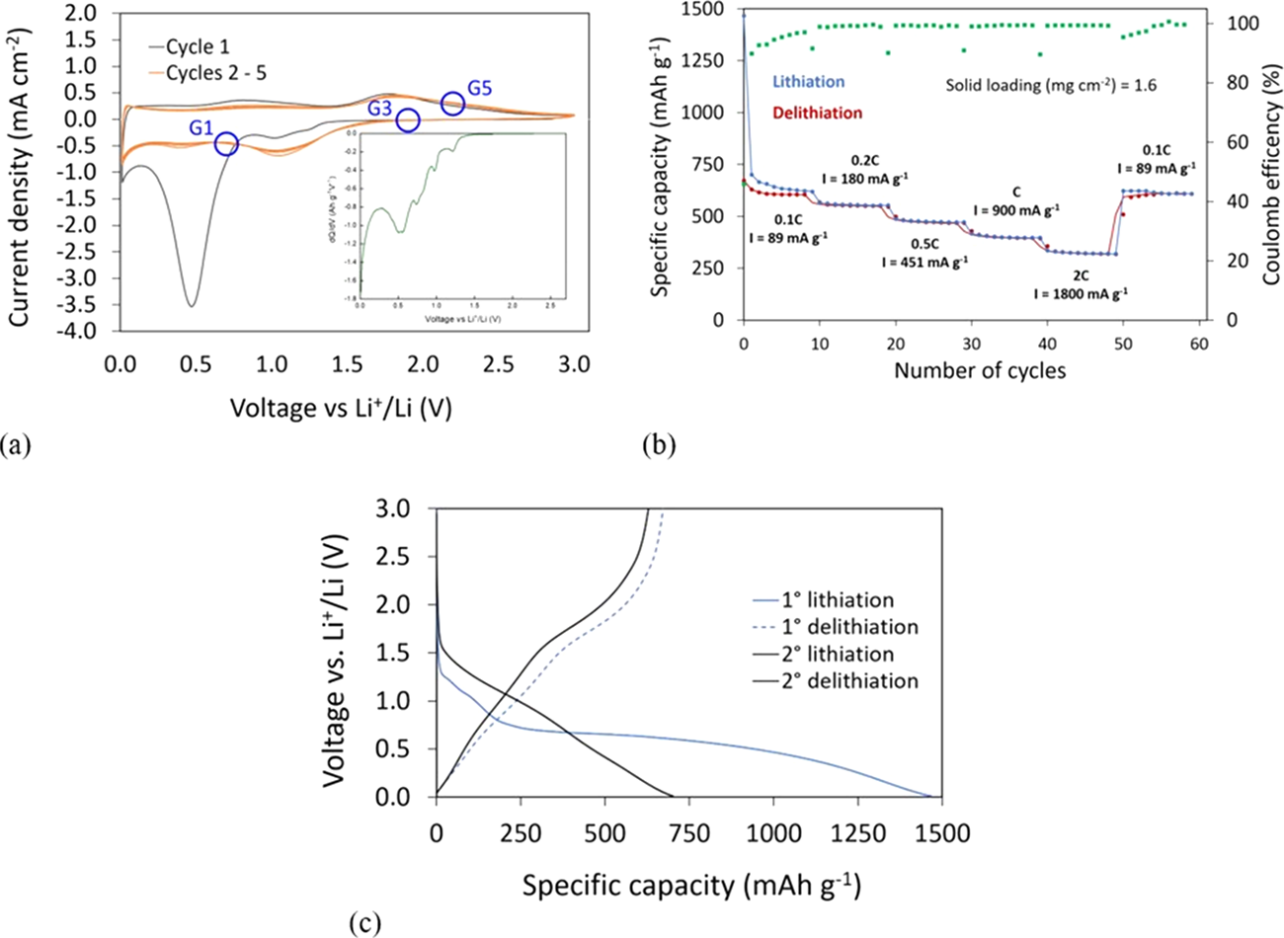
Lithiation/delithiation of the TM-HEO/1.0 M LiPF_6_ EC–DMC (1:1 v/v)/Li cell in the voltage range 0.01–3 V. (a) Cyclic voltammograms at 0.2 mV s^–1^. The inset reports the differential capacity during the first lithiation step. (b) Galvanostatic cycling at different current densities. (c) Voltage profiles for the 1st and 2nd cycle.

The electric conductivity of Mg_0.2_Co_0.2_Ni_0.2_Cu_0.2_Zn_0.2_O and the four-cation compositions was measured by means of electrochemical impedance spectroscopy on sintered pellets between 100 kHz and 1 Hz at 50 mV amplitude in the temperature range 60–900 °C. The resulting Arrhenius plots are reported in Figure S2. As noticeable from the graphs, conductivities of about 10^–7^ S cm^–1^ are obtained at 60 °C for both the high (five cations) and medium (four cations) entropy oxides. These values exceed what is generally observed in the literature for pure NiO and CoO oxides and feel the positive combining effects already observed in the literature in the case of mixed crystals of NiO–CoO mixtures, in a molar composition of at least 25:75 mol %.^17^

### 2.2 Anode Preparation

The anode slurry was prepared using 70 wt % active material (TM-HEO), 20% conductive carbon black (Timcal-Imerys, Super C65), and 10% binder (poly(vinylidene fluoride), PVDF). The solid content of all slurries was kept between 26 and 28 wt %. HEO and carbon were initially mixed in zirconia jars by a planetary ball mill at 150 rpm for 10 min, followed by a 5 min break and another 10 min of milling in the reverse direction. Subsequently, it was dispersed in the PVDF solution in *N*-methylpyrrolidone (NMP) to obtain the slurry, which was cast on a copper foil using a doctor blade with a wet thickness of 100 μm. The anode was finally dried under vacuum at 80 °C before the cell assembly.

The same experimental protocol was used to prepare the anodes based on the systems (CoCuMgNi)O and (CoCuNiZn)O.

A similar procedure was finally followed to assemble the cell for the operando investigation, except for the current collector. In this case, carbon cloth was used instead of copper foil to avoid any interference of Cu from the collector in the XAS spectra. The resulting mass loadings were 1.6 and 7.5 mg cm^–2^ for the Cu-based and carbon cloth-based cells, respectively.

### 2.3 Cell Assembly

Round disc electrodes with 10 mm diameter were cut and dried at 100 °C in vacuum for 12 h. All electrochemical measurements were performed in a Swagelok-type three-electrode cell assembled in an Ar-filled glovebox (H_2_O and O_2_ <0.1 ppm). Metallic Li was used as both reference and counter electrodes. Electrodes were separated with a Whatman glass fiber separator, imbibed by the liquid electrolyte consisting of 1 M LiPF_6_ solution in ethylene carbonate–dimethyl carbonate (EC/DMC, 50:50 vol %) (200 μL). Cells were galvanostatically cycled using a battery tester from 3.00 to 0.01 V (Bio-Logic BSC-810). A theoretical capacity of 900 mAh g^–1^ was considered, in agreement with what is typically adopted in the literature for similar TM-HEO-based anodes.^13^ The cyclic voltammograms were measured using an electrochemical interface (Solartron 1287) at a scan rate of 0.2 mV s^–1^. All potentials reported refer to the Li^+^/Li couple.

Two cells were assembled for each system (TM-HEO, (CoCuMgNi)O and (CoCuNiZn)O) and analyzed simultaneously to check the measurement reproducibility.

Electrochemical impedance spectroscopy was performed to determine the bulk conductivity of the active materials and the measurement of cell impedance. Nyquist plots were collected in the frequency range between 1 kHz and 0.1 Hz and a voltage amplitude of 50 mV.

### 2.4 Ex Situ and In Operando and X-ray Absorption Spectroscopy Measurements

Ex situ XAS experiments were carried out on HEO–1.0 M LiPF_6_ EC–DMC (1:1 v/v)/Li, frozen at three different potentials, 0.7, 1.9 (in the cathodic side), and 2.2 V (in the anodic one), corresponding to the main CV peaks. Before measurements, the cells were disassembled and the anode was isolated, washed with ethanol, and dried. Subsequently, the Cu current collector was removed, and the electrode powder was mixed with cellulose (at a ratio of 20:1 cellulose/powder) and finally pressed at 5 ton for 1 min to obtain compact pellets.

XAS data were acquired on B18, the Core XAS beamline at Diamond Light Source. Radiation from the bending magnet source was collimated and partially focused with Pt-coated Si mirrors to obtain a beam footprint on the sample of approximately 1 × 1 mm^2^. A double-crystal monochromator was equipped with Si(111) crystals, and higher-order harmonics were removed with the insertion of Pt-coated harmonics rejection mirrors placed at 7 mrad to the incoming beam direction. This setup, with Diamond Light Source operating at 3 GeV and 300 mA, provides ∼5 × 10^11^ monochromatic X-ray photons/s on the sample with resolution Δ*E*/*E* close to the nominal value for Si(111) of 1.4 × 10^–4^. XAS spectra were acquired in the continuous scan mode, with acquisition intervals of 0.25 eV over a range of 1000 eV across the K absorption edges of Co, Ni, and Cu.

Several continuous scans of durations of approximately 3 min each were acquired and averaged. Data were acquired in the transmission mode, with three ionization chambers partially filled with 170 mbar (N_2_) and 270 mbar (Ar) for the first, second, and third chambers, with pressures adjusted to absorb approximately 10 and 70% of the beam intensity at the Co K-edge. Beam energy calibration was ensured through the simultaneous acquisition of XAS spectra from metal foils of the same elements, inserted between the second and third acquisition chambers.

The operando XANES spectra were measured at the XAFS beamline operating at the Elettra synchrotron radiation facility in Trieste, Italy. The spectra were acquired at room temperature at the Co, Ni, and Cu K-edges using a silicon drift detector in the fluorescence mode. The ring current and energy were 200 mA and 2.4 GeV, respectively. A Si(111) double-crystal monochromator was used ensuring high-order harmonic rejection by detuning the second crystal. A water-cooled, Pt-coated silicon mirror was used to obtain vertical collimation of the beam. Spectra at the end of the charging process were also collected at the Zn K-edge, in the same experimental conditions. EXAFS spectra of the pristine sample were also collected at the Co, Ni, Cu, and Zn K-edges in the transmission mode. To this aim, an appropriate amount of sample to give one absorption jump in logarithmic units at each of the edges was weighted and thoroughly mixed with cellulose in an agate mortar and pestle and then pressed to pellets. The EXAFS spectra were extracted from the raw data by the Athena code.^18^ The operando electrochemical cell was an ECC-Opto-Std test cell (EL-CELL) equipped with an ECC-Opto Polymide window for X-ray experiments. XAS spectra were coupled to electrochemical measurements by the following protocol: (1) GITT performed at a constant current pulse of 1 h and a relaxation time of 10 min, (2) potential electrochemical impedance spectroscopy (PEIS) in the frequency range from 10 kHz to 1 Hz with an amplitude of 10 mV, and (3) equilibration at OCV. The XAS spectra were collected during the GITT and equilibration steps. The resulting GITT is reported in Figure S3.

For the X-ray absorption near-edge structure (XANES) analysis, the spectra were processed by subtracting the smooth pre-edge background fitted with a straight line. The spectra were then normalized at unit absorption at 300 eV above each edge, where the EXAFS oscillations are small enough to be negligible. Linear combination fittings of the postmortem XANES spectra were performed by means of the Athena code.^18^ A typical fit included more than 200 data points and no more than 2 variables. Successive multivariate curve resolution (MCR) and partial component analysis (PCA) of the whole sets of the operando XAS data^19^ confirmed the linear combination fitting results, taking into account the differences between the two data sets.

### 2.5 XRD and SEM

Powder X-ray diffraction was carried out by using a D8 Advance diffractometer (Bruker). SEM and energy-dispersive X-ray spectroscopy (EDS) were performed using a Tescan Mira3XMU microscope operated at 20 kV and equipped with an EDAX EDS analysis system. The samples were coated with a carbon thin film using a Cressington 208 carbon coater.

## 3 Results

### 3.1 Electrochemical Performances

The electrochemical performance of Mg_0.2_Co_0.2_Ni_0.2_Cu_0.2_Zn_0.2_O as the anode in Li-ion cells was studied using metallic Li as the counter electrode. Figure 1a shows the cyclic voltammetry plots of TM-HEO by sweeping the voltage from 0.01 to 3.0 V vs Li^+^/Li at a scan rate of 0.2 mV s^–1^.

According to the literature,^7,20−22^ the cathodic peaks at a voltage lower than 1.6 V are attributed to the lithiation process with the consequent oxide decomposition to the corresponding metals (like Ni, Co, and Cu) and Li_2_O. The intensity of the signal at 0.4 V is significantly reduced under further cycling due to irreversible phenomena during the first lithiation step, such as incomplete Li^+^ extraction from Li_2_O and the solid electrolyte interphase (SEI) formation. In contrast, two broad and not intense oxidation signals peak at around 0.8 and 1.9 V, which are possibly due to delithiation of Li_2_O and consequent phenomena, such as oxidation of M to MO^20−22^ and/or Li/M alloying.^7,23^

In the first cycle, at least three signals between 1.3 and 0.8 V are present, which can be associated with a multistep reaction leading to a Cu^2+^/Cu^+^ solid solution, to the formation of Cu_2_O phase, and to the final decomposition producing metallic Cu.^21,24^

Figure 1b shows the galvanostatic cycling and some voltage discharge–charge profiles at 0.1 C of the TM-HEO–Li cell between 0.01 and 3 V vs Li^+^/Li. The profiles and the related differential capacity plots collected at higher C rates are reported in Figure S4a,b. Except for the low current region 0.1 C (both during the first cycles and at the end of the rate performance experiments), the Coulombic efficiency is very close to 1 and the specific capacity reaches values higher than 300 mAh g^–1^ at 2 C, in good agreement with the literature.^13,14^ On the other hand, the efficiency increase with the C rate is an expected phenomenon. At low current densities, slow parasitic reactions (e.g., electrolyte consumption in the case of uncomplete SEI) may occur at the expense of the charge. Especially during the first cycles at 0.1 C, the delivered capacity is halved during the initial two cycles. Indeed, the first lithiation and delithiation capacities are 1325 and 700 mAh g^–1^, respectively, with a Coulombic efficiency of about 53% (see Figure 1c). This specific value is associated with the formation of an SEI layer and the structural and morphological changes taking place upon (de)lithiation.^25^ As observed in the CV plots, the profiles of the second cycle are quite different from those of the first one, with less evident plateaus, suggesting a different reaction mechanism in the case of further cycling.

### 3.2 Spectroscopic Investigation: In Operando and Postmortem XAS

Aiming at understanding the actual working mechanisms of Mg_0.2_Co_0.2_Ni_0.2_Cu_0.2_Zn_0.2_O TM-HEO as the anode material for Li batteries, we prepared three samples by freezing the HEO-based Li-ion cell at three different voltages (0.7, 1.9, and 2.2 V) during the second lithiation/delithiation cycle (Figure 1a), two in the cathodic (labeled in the following as G1: *V*_c_ = 0.7 and G3: *V*_c_ = 1.9 V) and one in the anodic (labeled in the following as G5: *V*_a_ = 2.2 V) sides, respectively: the potential position of these samples is highlighted with blue circles in Figure 1a. Such values are the end voltages of the main peaks observed in the cyclic voltammograms and were chosen to identify which ion in Mg_0.2_Co_0.2_Ni_0.2_Cu_0.2_Zn_0.2_O is involved in the reduction/oxidation processes.

Such three samples were, in fact, subsequently investigated ex situ by XAS at the Co, Ni, and Cu K-edges. The application of a local probe such as XAS was dictated by the lack of crystalline order found at the end of the lithiation process; we choose to investigate the Co, Ni, and Cu K-edges since, according to the standard reduction potentials, these metals are more easily reducible than Zn and Mg and therefore are more likely to participate in the early stages of the lithiation process. In addition, as noted above, the signals in the CV between 1.3 and 0.8 V were assigned to a multistep reduction involving Cu, and this needed further investigation. This is demonstrated by Figure S5, where an XRD pattern of the Mg_0.2_Co_0.2_Ni_0.2_Cu_0.2_Zn_0.2_O anode material at the end of the process is shown. The pattern shows no clear diffraction effects, thus demonstrating that the lithiation process produces an amorphous material.

**Figure 2:**
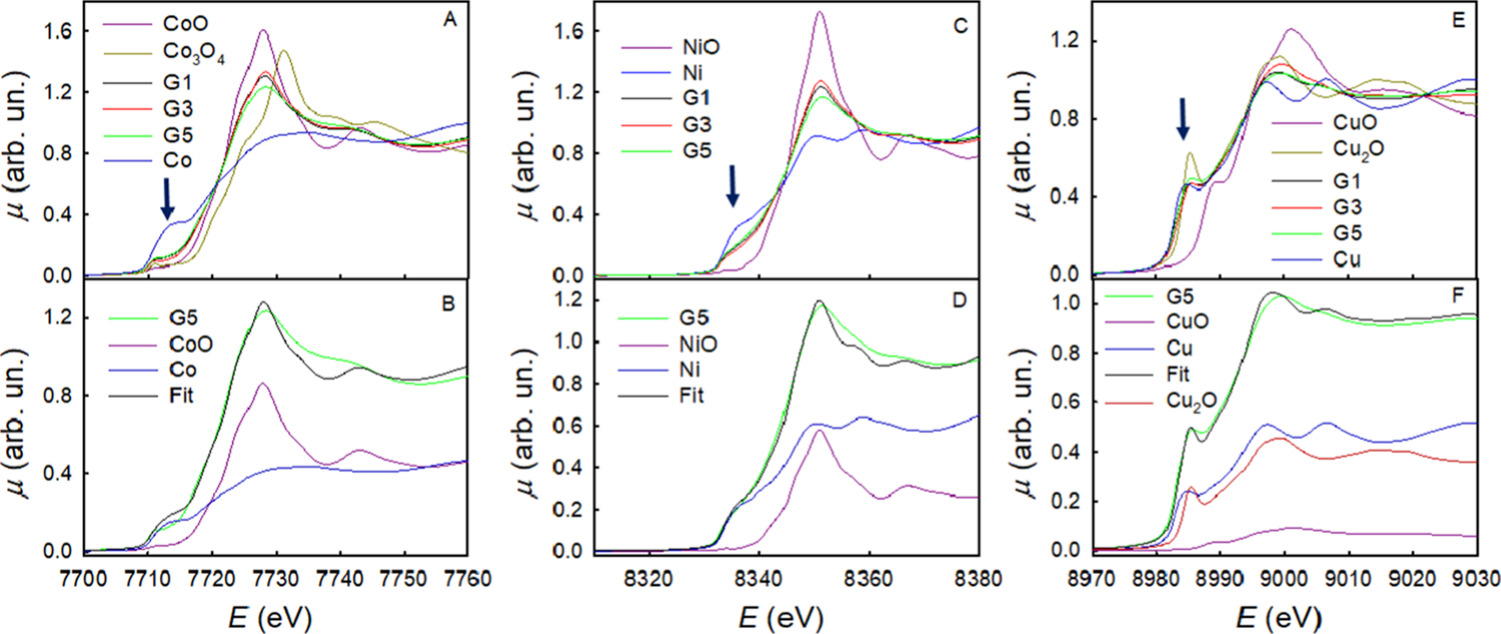
Co, Ni, and Cu K-edge XANES spectra for samples G1, G3, and G5 (A, C, E). The spectra of Co, Ni, and Cu oxides, as well as of Co, Ni, and Cu metals, are also shown for reference: the blue arrows mark the position of the metal shoulders on each of the absorption edges. (B, D, F) Fits of the spectra of the G5 sample at the Co, Ni, and Cu K-edges, respectively, with linear combinations of the spectra of the reference compounds, which are also shown in the figure weighted by the coefficients of the linear combinations.

Figure 2A,C,E shows the XANES spectra of the G1, G3, and G5 samples at the Co, Ni, and Cu K-edges, respectively. The spectra of CoO, NiO, CuO, and Cu_2_O standards are also shown for comparison, along with the spectra of metallic Co, Ni, and Cu. For each element, the edge energy position is determined by the binding energy of the 1s electrons, which in turn is controlled by the Coulombic potential. This can be screened by the outer (valence) electrons: as a result, the edge shifts at higher energies with increasing oxidation state. At the Co K-edge, the edge energy position of all samples is similar to that of CoO. However, for all of the samples, a tail at lower energy appears. This is at the same energy position of the hump at 7712 eV in the spectrum of metallic Co. In transition metals, this structure is due to the excitation of 1s core electrons to empty states in the conduction band. Looking at the Ni K-edge spectra, quite similar results are found: the edge energy position of all of the samples closely matches that of NiO, but a large spectral intensity is found in the hump region at ca. 8335 eV of Ni metal. In this case, this additional spectral intensity is larger than that found at the Co K-edge. At the Cu K-edge, the spectra of all of the samples are in an energy position that closely resembles that of Cu_2_O; however, also, in this case, some further spectral intensity is found in the spectral region at ca. 8982 eV, where metallic Cu has its first maximum.

These results point toward the presence of Co(II), Ni(II), and Cu(I) in all of the samples, together with the presence of Co, Ni, and Cu in a metallic state. This is consistent with the EXAFS spectra and their Fourier transforms shown in Figure S6. The presence of both Cu(I) and Cu(0) is in agreement with the equilibrium Cu/O phase diagram.^26^ To test this hypothesis, the spectra of the G5 sample, taken as a representative of all of the other samples, were fitted for all of the edges with linear combinations of the spectra of the corresponding metals and oxides. The results are shown in Figure 2B,D,F for the Co, Ni, and Cu edges, respectively; the results for samples G1 and G3 are shown in Figure S7. The weights for the linear combinations are shown in Table 1 (Table S1 for the G1 and G3 samples). The quality of the fits, as measured by the respective R indexes, is quite good in all of the cases: the best agreement was found for the Cu K-edge, where the quality of the fit was reasonably improved by adding to the linear combinations also the spectrum of CuO. This is attributed to the fact that at the end of the lithiation process, a high degree of amorphization was found (see below), which is highly suggestive that the local structure of Co is close to that of the liquid and therefore to that of the metallic fcc phase.^27^ In the fits, we were obliged to use the experimental XANES of hcp Co: therefore, the fact that the worst agreement is obtained in the case of Co is fully sensible. The fraction of metallic Ni is larger than both the fractions of metallic Co and Cu; this may be due to the more easy reducibility of Ni(II) when compared to Co(II) and combined with the fact that Cu(II), which is the more reducible of the three metals, reduces to both Cu(I) and Cu(0).

**Table 1:** Linear Combination Fitting Results of the Co, Ni, and Cu K-edge XANES Spectra of Sample G5, Expressed as Mole Fractions^a^

	Co K	Ni K	Cu K
Co	0.464		
CoO	0.536		
Ni		0.665	
NiO		0.335	
Cu			0.518
CuO			0.074
Cu_2_O			0.409

a The fit quality is expressed by the R index, equal to 0.0099, 0.0077, and 0.0016 for the Co, Ni, and Cu K-edge spectra, respectively.

The worst agreement obtained at the Co K-edge may be due to the fact that metallic Co is one of the few metals having a hexagonal close-packed crystal structure. If a metallic alloy is formed during the Li charge–discharge cycles, the crystal structure of the alloy may reasonably differ from hcp. In any case, it is apparent that a large amount of Co, Ni, and Cu is in the metallic state. This amount is the largest for Ni and the smallest for Co. In addition, almost all Cu(II) appears to be reduced, nearly half of which to Cu(I) oxidation state.copper amount to the Cu(I) oxidation state.

All of these results point toward the fact that the conversion reaction forming metals and Li_2_O is the actual working mechanism for the HEO as the anode. However, the reaction is not completed and totally reversible. In the cathodic region, the reduction of the oxides does not fully occur as the oxidation of metals in the anodic domain, but a coexistence of M and MO is clearly noticeable. Furthermore, both pieces of evidence that the behavior of the three transition metals is different and that copper is sequentially reduced to Cu(I) and Cu(0) ask for a more detailed investigation.

To have a better understanding of the working mechanisms of the HEO anode, we undertook an operando XAS investigation on the first lithiation cycle by performing XANES measurements at the Co, Ni, and Cu K-edges on a working cell at different values of capacity delivered by the cell during the lithiation step (*Q*).

**Figure 3:**
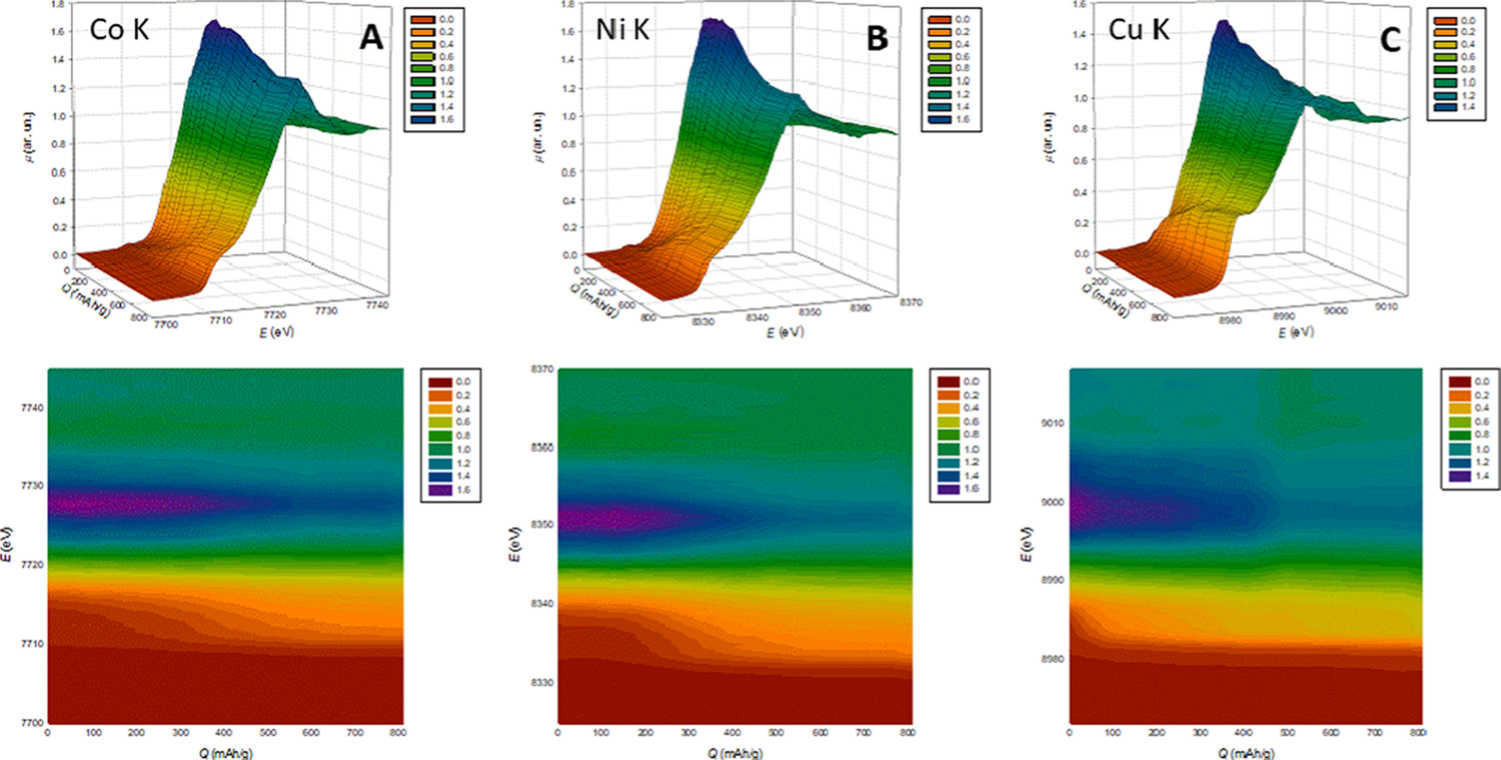
Co (A), Ni (B), and Cu (C) K-edge XANES spectra in a working HEO battery as a function of the delivered capacity during the lithiation, as 3D plots (upper panels) and contour plots (lower panels). The contour plots refer to the same data as the 3D plots and are shown for the sake of better clarity.

The results are shown in Figure 3. As in Figure 2, the reduction of metals is made evident by the appearance of a shoulder at low energy on the edge, i.e.*,* at ca. 7712, 8335, and 8982 eV for Co, Ni, and Cu, respectively, as marked by the blue arrows in Figure 2A,C,E. It is clearly apparent that the three transition metals have distinct behaviors.

The reduction of Cu is the first to be detected, clearly evident at *Q* = 45 mAh g^–1^, occurring in the voltage range between 1.2 and 0.9, in very good agreement with the two cathodic peaks observed during CV and galvanostatic cycling (Figure 1). At *Q* = 135 mAh g^–1^ (*E* ≅ 0.8 V), the first traces of metallic Co become visible, while Ni metal starts to appear at *Q* = 180 mAh g^–1^ (*E* ≅ 0.7 V). In addition, the amounts of reduced metals are very different. For *Q* > 495 mAh g^–1^ (*E* < 0.5 V), virtually all Cu is in the metallic state, while a relevant fraction of the oxide is present for Co and Ni even at the end of the charging process (i.e., when *Q* is ca. 90% of the theoretical capacity of the battery). The oxide molar fractions are 0.4(1) and 0.3(1) for Co and Ni, respectively, as estimated by fitting the pertinent spectra obtained at the end of the process with linear combinations of the spectra of the metals and those at OCV.^19^

**Figure 4:**
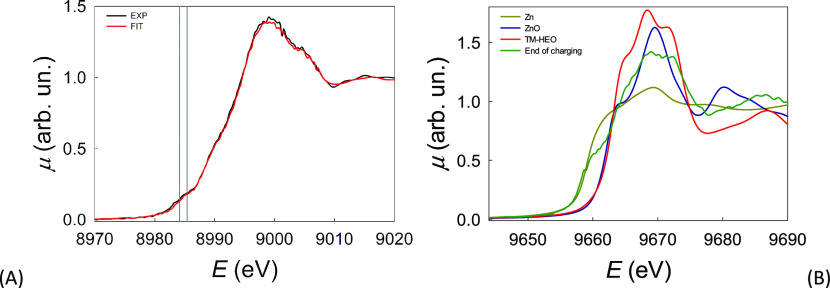
(A) Cu K-edge XANES spectrum at *Q* = 45 mAh g^–1^ (black line) and the fit using a linear combination of the spectra at OCV of Cu and Cu_2_O (red line). (B) Zn K-edge XANES spectrum at *Q* = 800 mAh g^–1^ (green line). The spectra of Zn, ZnO, and pristine TM-HEO are also shown for reference.

The initial lithiation steps are particularly interesting, as only Cu appears to be involved in the reaction. We used the spectrum at the Cu K-edge at *Q* = 45 mAh g^–1^ to have a closer look at the first steps of the reaction. At *Q* = 45 mAh g^–1^, the spectra at both the Co and Ni K-edges remain unchanged, thus proving that the early steps of the reaction involve Cu only. The Cu K-edge spectrum was fitted using linear combinations of the spectra at OCV of metallic Cu and Cu_2_O. The results are shown in Figure 4A. It is worth noting that both Cu and Cu_2_O are needed to obtain a good match of the shoulder at ca. 8985 eV. This result, which was confirmed by MRC and PCA analyses of the whole set of the operando XAS data,^19^ is direct proof that the first step of the reaction is the reduction of Cu(II) to Cu(I): we here note incidentally that the MRC and PCA analyses of the whole set of the operando XAS data^19^ also confirmed the results of XANES fittings for samples G1, G3, and G5, with the fact that the operando data are taken on the first lithiation step, while the postmortem samples refer to the second CV cycle. We also note again that the Co and Ni K-edges remain almost unchanged up to ca. *Q* = 200 mAh g^–1^ (*E* < 0.7 V), i.e., when ca. 60% of Cu is still present as Cu(II) oxide, thus implying that the rock-salt structure of HEO is quite robust toward the reduction of Cu. This is due to the high configurational entropy adding additional stabilizing terms to the Gibbs free energy.^28^ However, for *Q* > 400 mAh g^–1^, we observe a strong reduction in the amplitude of the main edge peaks (white lines, WLs) at both the Co and Ni K-edges, at ca. 7728 and 8351 eV, respectively. In addition, the Cu K-edge seems to be affected as the WL at 8998 eV shows the final decrease for 400 < *Q* < 600 mAh g^–1^. We remark here that XANES is a powerful probe of the local order around the photoabsorber: the abrupt change in the WL amplitude at *Q* > 400 mAh g^–1^ at both the Co and Ni K-edges is therefore related to an abrupt change in the local order around both Ni and Co; for the same reason, also, the local order around Cu is affected. We also observe that the WL amplitudes are never recovered and the XRD pattern at the end of the lithiation process indicates complete amorphization of the material. The combination of these facts allows stating that at *Q* > 400 mAh g^–1^ the TM-HEO structure collapses.

### 3.3 Morphological and Microstructural Investigation

The morphology of the as-prepared TM-HEO and the HEO-based anode after the galvanostatic cycling was investigated by SEM-EDX analysis. As shown by electron microscopy images (Figure S1a), HEO particles smaller than 5 μm are obtained by preparing the high-entropy oxide through a solid-state reaction at high temperatures. The microsize of the active material may be responsible for the complex electrochemical mechanisms,^7^ proposed by considering the results obtained from XAS investigation.

**Figure 5:**
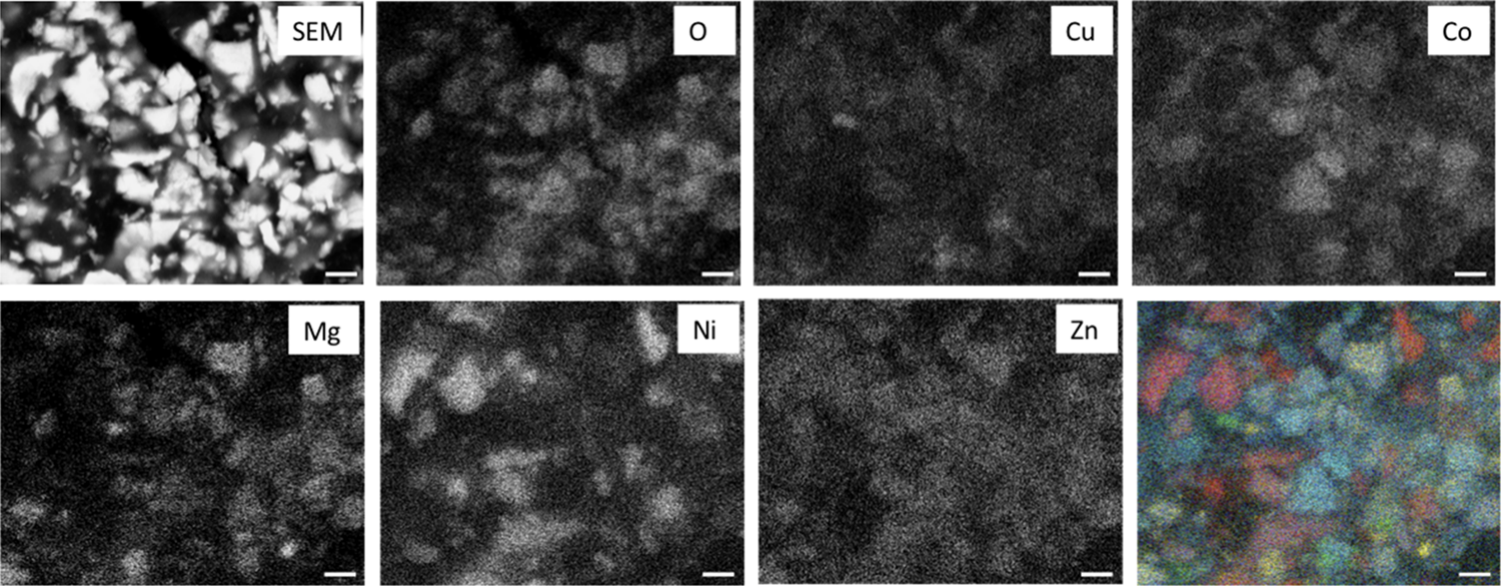
Postmortem SEM image of the TM-HEO anode (upper left corner) and EDS mapping images of the same region for the component cations. The false-color image in the lower right corner has been obtained by overlapping all of the EDS maps. In this image, blue represents Zn, yellow is Mg, red is Ni, green is Cu, and cyan is Co. The white bar present on all images is 2 μm in length.

The postmortem SEM image (Figure 5) shows the presence of irregular grains, presenting an average dimension of about 2 μm. Surprisingly, the EDS analysis gives evidence of a quite inhomogeneous distribution of the component cations. Each grain presents, indeed, a dominant distribution of one of the cations. This can be observed in the maps relative to the distribution of each metal, but it appears to be even clearer in the false-color image obtained by overlapping all of the maps; in addition, it is clearly seen by the oxygen map that some grains are oxygen-free or have a low oxygen concentration, and therefore, they can be safely assigned to a metallic phase. Considering that, before the electrochemical treatment, the grains were uniform in composition (Figure S1c and related discussion in Section 2), a possible explanation must consider that galvanostatic cycling collapses the HEO structure with demixing of the original oxide. In this scenario, each grain ends up presenting an inhomogeneous distribution of the cations, resulting in an apparent different composition.

Although we have no definitive explanation of this finding, we here note that the lithiation sequence outlined above can give a potential explanation of the observed formation of large grains with segregated composition when the dissolution of the transition-metal ions in the electrolyte (namely, LiPF_6_ in organic carbonates) is taken into account. This phenomenon is well known in the case of lithium-ion and lithium batteries, both for anodes and cathodes, still representing a critical challenge for the development of next-generation batteries.^29^ When Cu starts to reduce, the first Cu(II) cations involved in the reduction are those present in the electrolyte solution. This reduction forms the first nuclei of metallic Cu and lowers the amount of Cu(II) in the solution: additional Cu(II) will then dissolve to restore the equilibrium concentration in the electrolyte. This dissolution–reduction sequence allows the metallic nuclei to grow. When Ni(II) also starts to be reduced, a lot of Cu is already in the metallic state, outside the HEO structure; therefore, grains of metallic Ni can be formed via the same mechanism. This rationale can be extended also for the other metals. The final result is the segregation of the metals at the end of the charging process, as shown by the EDX analysis. The compositional heterogeneity is not so rare in the case of compounds including transition metals as in the cathode systems or metal oxides for anodes. Several articles report on how the compositional and/or structural inhomogeneities of a composite electrode could affect the rate performances of a lithium battery, leading to the loss of capacity and local overcharge or discharge or unsafe conditions, for instance, loss of oxygen in the case of transition-metal oxides at high SOCs. Therefore, an understanding of the formation mechanism during the lithiation/delithiation steps is fundamental from a practical point of view. Focused studies were discussed in the literature investigating the state of charge heterogeneity by means of several techniques, as in situ X-ray diffraction (XRD), ex situ micro-Raman mapping or synchrotron X-ray imaging, and spectroscopy, of several TM oxides.^30−32^ Further insights can be obtained using nanoscale full-field X-ray spectro-microscopy, as recently demonstrated by Wei and co-workers.^33^

## 4 Discussion

**Scheme 1:**
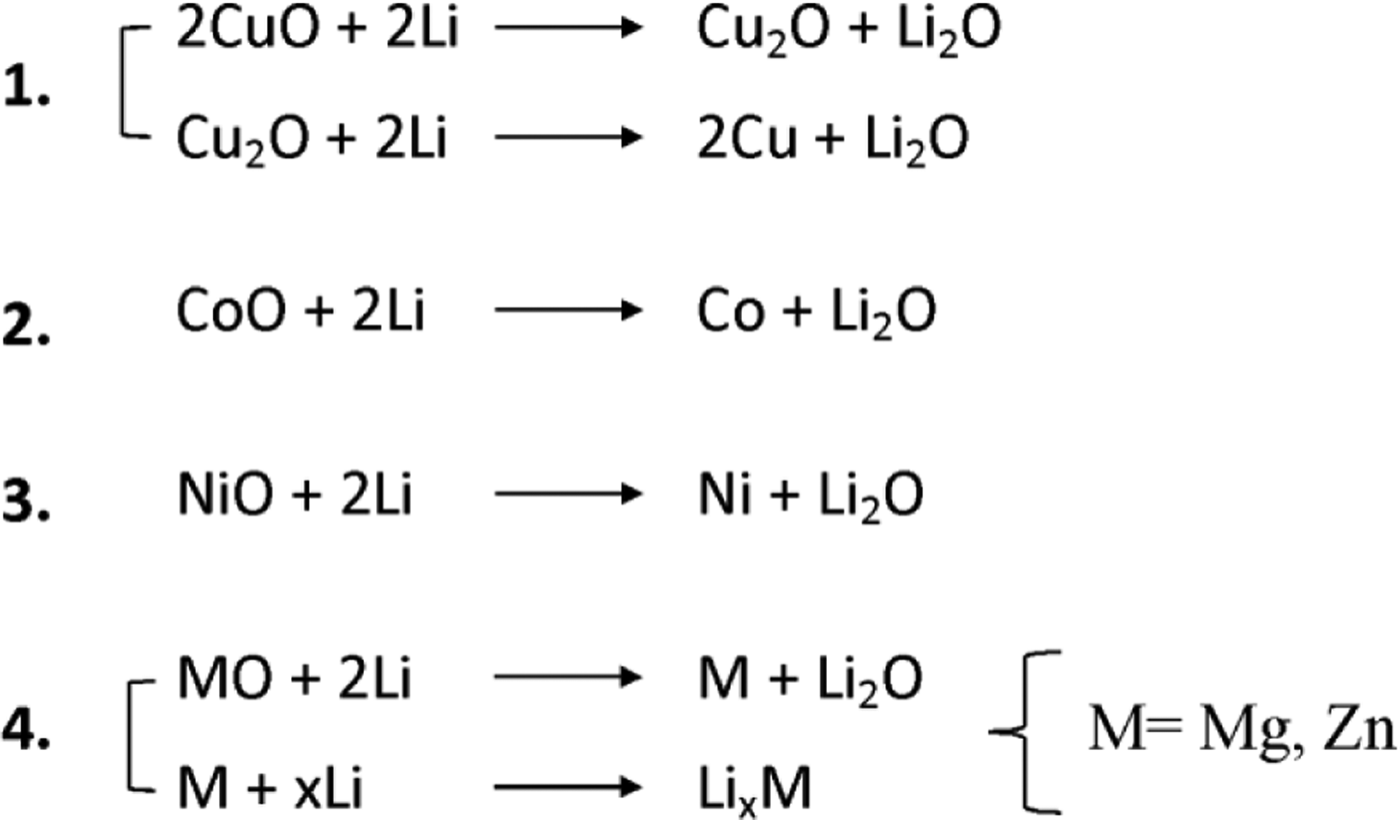
Multiple-Step Mechanism Proposed for TM-HEO Lithiation

Basically, the lithiation of TM-HEO induces a very complex mechanism involving a two-stage process: (i) the conversion of some cations as Cu^2+^, Co^2+^, and Ni^2+^ and (ii) the conversion and subsequent alloying/dealloying of Mg^2+^ and Zn^2+^, as sketched in the following Scheme 1

Cu is the first element involved in the reduction through a multistep reaction starting at 1.2 V, which leads to Cu^+^ and, finally, to Cu^0^, storing Li_2_O.

At potentials lower than 1.2 V, further Cu reduction gives way to the conversion reaction. The HEO cubic structure remains intact for ∼60% of lithiation delivered capacity (see Figure 3), thanks to the matrix-stabilizing effects of ZnO and MgO at these potentials, in fair agreement with the literature.^12^ Such a result well proves the beneficial role of the configuration entropy in enhancing the stability of the HEO rock-salt structure during the redox phenomena, even if not in the whole explored potential range.

By further decreasing the voltages, the Co^2+^ and Ni^2+^ reduction takes place, as evidenced by the appearance of the shoulders at ca. 7712 eV at the Co K-edge and at ca. 8335 eV at the Ni K-edge. In addition, the intensity of the main peak at both the Co and Ni K-edges considerably lowers. Taking into account that XANES is sensitive to three and four body distribution functions around the photoabsorber, this is unequivocal evidence that Co and Ni are in a chemical environment that is different from that of TM-HEO. While it is possible that MgO and ZnO still form a metastable solid solution in this potential region, the rock-salt structure collapses, leading to a mixture of Co, Ni, and Cu and the corresponding oxides, MO, in a molar ratio as reported in Table 1. The conversion reaction is therefore not completed, and the consequent decomposition of Li_2_O to reconvert metals into the TM-HEO rock-salt structure is not reversible. Indeed, metals (Ni, Co, Cu) and the corresponding oxides (CoO, NiO, and Cu_2_O) remain segregated and are electrochemically inert under further cycling: this is demonstrated by the very close similarity in the spectral shape for samples G1, G3, and G5 at the Co, Ni, and Cu K-edges, both concerning XANES and EXAFS. In addition, the lack of long-range order demonstrated by XRD and the large degree of cation segregation demonstrated by EDX, both at the end of the lithiation cycle, clearly support the conclusion of a large degree of irreversibility.

This spectroscopic result is in very good agreement with the SEM analysis, showing very large aggregates of M and MO particles, as discussed in detail in the previous section. The nonreversibility of the conversion reaction is reasonably related to the morphology of the products resulting from the first lithiation step. It is well known, in fact, that in the case of conversion oxides, the decomposition of the electrochemically inert lithium oxide to Li and MO is allowed only in the presence of metal nanosized nuclei, which are able to catalyze such processes.^22^

Concerning Zn, the XAS results show that the Zn K-edge spectra (Figure 4B), collected before and after the electrochemical experiment, show dramatic changes. Indeed, the spectrum of pristine TM-HEO shows a perfect agreement with that of cubic ZnO with the rock-salt structure.^34^ On the contrary, at *Q* = 800 mAh g^–1^, a marked shoulder is detected at ca. 9660 eV, which corresponds to the shoulder of the spectrum of metallic Zn and is due to the electronic transition from the 1s orbital to empty states in the conduction band: this has the obvious meaning that at the end of the charging process, a significant fraction of Zn is found in the metallic state, in contrast with what is reported by Sarkar et al.^12^ However, the global XANES spectral shapes at the Zn K-edge of pure metallic Zn and of the sample at the end of the lithiation process are considerably different. Bearing in mind that XANES is sensitive not only to the electronic structure as projected onto the photoabsorber but also to the actual atomic local structure around the photoabsorber, this is strongly suggestive that Zn is indeed not present as pure metallic Zn and, thus, that an alloy (possibly with Li) is formed. Moreover, the presence of a peak in the absorption edge at the same energy as the main peak of ZnO points to the presence of a significant amount of zinc in an oxidic phase.

However, the spectroscopic study, showing incomplete and irreversible conversion, does not fit the electrochemical investigation, which, in contrast, shows high specific lithiation/delithiation capacity and rate capability, even at high current densities (see Figure 1).

Recently, the reversible capacity obtained in similar electrodes was attributed to a mix of pseudocapacitive and faradic contributions, the latter due to the reversible conversion reaction^35^
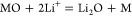
Such data treatment arises from the current peak, *I*_p_, analysis of the cells in the CV plots collected at different scan rates, υ, which follows a power law as in the following equation
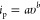
where *b* is the slope of the linearized log/log equation, which could provide insight into the charge-storage mechanism. *b* ranges between 0.5, indicating full faradic intercalation controlled by semi-infinite linear diffusion, and 1, representing a surface capacitive charge storage, free of diffusion control. We analyzed the *b* slope in the HEO CV plots collected between 0.1 and 1.0 mV s^–1^, obtaining values of about 0.8 (see Figure S8), which, in principle, could confirm the mixed faradic/pseudocapacity contribution reported in the literature. However, such a hypothesis is not convincing. This linear relationship as well as the fitting parameter *b* strictly, in fact, rules only at the specific potential of the peak current, and accordingly to Bard and Faulkner,^36^ the *b* fitting parameter is equal to 1 only at the potential corresponding to peak current for diffusion-controlled reactions. For this reason, such a procedure has not been validated right now by a more complex treatment, although sometimes used in the literature, and could lead to misleading results, especially in the case of complex electrochemical phenomena.

It was also shown that the pseudocapacitive behavior is proportional to the BET surface area in samples milled for different times and becomes the most important source of charge storage for small particles (up to 90%). However, our spectroscopy measurements demonstrate the irreversibility of the oxide to metal conversion, leaving a mixture of metals and partially reduced oxide at the end of the first cycle, which are not further reconverted over cycling. Moreover, the observed particle size is quite large (Figure S1a). For all of these reasons, we do not believe that the pseudocapacitive contribution is relevant to justify the delivered capacity observed in such a system.

Another consequence of extra capacity could also derive from the reversible electron transfer involving the anions of the liquid electrolyte (e.g., carbonate) during the conversion step to form the corresponding metal salts.^37^ However, our XAS measurements provide spectra that are specific for the metal oxides, rather than for carbonates, which, in contrast, are described by significantly different spectroscopic signals.

It is then more reasonable discussing these reversible performances in terms of a combination of alloying/dealloying phenomena between two binary systems: (i) Li and Zn and (ii) Li and Mg, whose kinetics (especially in the case of MgO) could be improved by the synergistic catalytic effect of metallic multielements (maybe nanosized), segregated during the first lithiation step. It is well known, for instance, that ZnO is an anode material based on the alloying/dealloying reaction since the respective metal can form alloys with Li, such as LiZn, at potentials lower than 0.7 V. Therefore, large reversible capacities are possible (overall theoretical capacity of 987 mAh g^–1 ^^25^).

In addition, Li shows very high miscibility in Mg and a wide range of possible binary systems, as shown by the phase diagrams reported in the literature.^38,39^ The resulting electrochemical process may occur through two steps: (i) reduction of MO to M and Li_2_O and (ii) formation of Li/M alloys (M = Zn, Mg). Taking into consideration the reversible charging/discharging processes obtained in the case of HEO-based cells and the molar fraction of ZnO and MgO (*x* = 0.2) in the TM-HEO, we may speculate that the average specific capacity of 615 mAh g^–1^ observed at 0.1 C can explain the concomitance of two phenomena: (i) LiZn alloy formation and (ii) and Li/Mg alloy formation with very high Li at.% (ranging around 80 at.%) (see SI Appendix A—Figure S15). The latter compositions are envisaged by the phase diagram of the magnesium–lithium system, which shows a wide range of stable Li solid solutions from 30 to 100 Li at.%, making possible the production of several binary alloys.^38^ The delivered capacity is also quite comparable with that reported in the literature for electrodes based on Li/Mg alloys, which show a specific capacity of ∼600 mAh g^–1^ in the case of alloys with Li contents of about 70 at.%.^40^

To experimentally prove the crucial role of both Mg and Zn in the electrochemical performances of the TM-HEO-based anodes, we investigated the cycling behavior of HEO-no Zn and HEO-no Mg. Both the pristine materials have rock-salt structures. However, in the case of the (CoCuMgNi)O system, the XRD pattern shows the presence of non-negligible CuO impurities (Figure S9a,b).

The galvanostatic cycling tests of both the samples have much worse performances than those observed for the TM-HEO-based anode. In the case of (CoCuMgNi)O (Figure S10a–e), the charge–discharge capacity delivered over cycling rapidly decreases during the first cycles to stabilize around 190 mAh g^–1^ at 0.2 C. Such a value is at least a factor of 2.5 lower than Mg_0.2_Co_0.2_Ni_0.2_Cu_0.2_Zn_0.2_O at a similar C rate. The voltage plateaus are well defined only during the first lithiation cycle (parts b and c) and this is clear evidence of highly irreversible phenomena. Such an aspect is better shown in Figure S10b, which reports the differential capacity for the first and second cycles. During initial lithiation, similar peaks to HEO are noted at comparable voltages, except for the Cu^2+^–Cu^+^ signal, which appears shifted at higher potentials (see below). In the second cycle, the peaks below 1.0 V, mostly ascribed to the reduction of Ni^2+^ and Co^2+^, disappear, and only the Cu signal is still evident, even if with lower intensity.

The (CoCuNiZn)O-based anode shows a significantly different mechanism (Figure S11a–e). In this case, the delivered capacity is even lower and drastically decreases close to zero during the first six cycles. The differential capacity shows the presence of just one main peak at around 1.1 V both in the anodic and cathodic sides, reversible only during the first lithiation cycle.

The comparison among the properties of Mg_0.2_Co_0.2_Ni_0.2_Cu_0.2_Zn_0.2_O, Mg_0.25_Co_0.25_Ni_0.2_Cu_0.2_, and Co_0.25_Ni_0.25_Cu_0.25_Zn_0.25_O suggests that both ZnO and MgO, in particular, the latter one, seems to be responsible for the capacity delivered by the TM-HEO-based anode.

Very complex phenomena occur, therefore, by changing the number of oxides and the type of metallic cations in the HEO. The entropy-stabilized oxide is a case on his own with its unique thermodynamics, different from the single oxide. Consequently, also, the electrochemistry changes with respect to the individual components. The reason lies in the configurational entropy, which significantly increases with the number of cations in the structure, strongly affecting the functional properties of the system, from the lithiation mechanism to the electrochemical potentials of the M^2+^/M couples. Regarding the latter aspect, this is particularly evident in the case of copper. Figure S12, for instance, compares the first lithiation cycle for all of the analyzed samples (e.g., TM-HEO, (CoCuMgNi)O, and (CoCuNiZn)O). The peak above 1.0 V, reasonably assigned to the reduction of CuO, is strongly influenced by the TM-HEO composition, ranging from 1.0 and 1.5 V, depending on the presence or absence of ZnO and MgO.

Another proof (even if indirect) of potential Li/M alloying comes from the poor electrochemical performances of TM-HEO obtained in the case of Na cells (see Figure S13a,b). From galvanostatic cycling, it is evident the serious inefficiency of the sodiation/desodiation process in this kind of system. A specific capacity significantly lower than 80 mAh g^–1^ (likely due mostly to the electrochemical activity of the carbonaceous binder fraction in the composite anode) and low Coulombic efficiency are obtained even at a low current density (0.1 C). Such worse performances may be likely ascribed, for instance, to the lower solubility of Na in solid M or of M in solid (Na),^41,42^ especially in the case of Mg (Figure S14), and then to the difficulty or impossibility of forming binary alloys, contrary to what occurs in the case of lithium.

A final comment concerns the lack of crystal order found at the end of the lithiation process. The molar configurational entropy for a high-entropy oxide, *S*_config_, with the rock-salt structure and no disorder on the oxygen site is
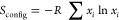
where *x*_*i*_ is the molar fraction of the *i* element on the cation site.^12^ The same relation also applies to an alloy of metals in a crystal structure with just one site, such as fcc or hcp. Thus, if an alloy of metals with the (say) fcc had been formed, no entropy increase would have been expected, and the lithiation process would have been reversible. The experiments show, on the contrary, that (i) the process is highly irreversible, indicating that the entropy production Δ*S*_*i*_ is large and positive, and (ii) the metal distribution shown by the EDX maps of Figure 5 is highly nonhomogeneous, indicating that the configurational entropy of mixing in the final lithiation reaction product is small. The only way that is left to the system to increase the entropy as required by the irreversibility condition (Δ*S*_*i*_ > 0) is to display no crystalline order at the end of the process.

## 5 Conclusions

Here, we proposed a new complex mechanism occurring during the lithiation/delithiation processes in high-entropy oxides based on transition metals with rock-salt structure as anodes for lithium-ion cells.

To this aim, we carried out both operando and ex situ XAS measurements on the system Mg_0.2_Co_0.2_Ni_0.2_Cu_0.2_Zn_0.2_O (TM-HEO), in combination with electrochemical and microstructural characterization. The spectroscopic results suggest that the redox reaction takes place through a multistep process depending on the TMs’ reduction potential. The whole process is incomplete and irreversible, leading to a mixture of M and MO with a variable molar ratio depending on the metal. In addition, contrary to what is supposed in the literature by XRD analysis, we prove that even ZnO takes part in the conversion mechanism since a significant fraction of Zn is found in the metallic state at the end of the charging process.

Despite the collapse of the rock-salt structure, a charge/discharge specific capacity higher than 600 mAh g^–1^ at 0.1 C has been delivered by the TM-HEO-based cell with the Coulombic efficiency very close to 1, in particular, at higher current densities. Taking into account the irreversibility of the redox mechanism, it is reasonable to ascribe such capacity to the alloying/dealloying reaction typical of anodes such as ZnO and MgO.

Even though the rock-salt structure of such a system is intended to collapse, it maintains up to 60% of charge, when the first traces of metallic Co are apparent (around 0.8 V), confirming the stabilizing effect of the configurational entropy.

However, our results clearly point out that Mg_0.2_Co_0.2_Ni_0.2_Cu_0.2_Zn_0.2_O is not still suitable for use as the anode in LIBs, despite the promising performances shown in the literature. Further strategies of phase stabilization should be therefore investigated, for instance, by properly modulating the important key factors, such as the type of metal, the number of cations, stoichiometry, and morphology, and also by exploring other structures, such as spinel, where a different lithiation/delithiation mechanism occurs.

We strongly believe that such a novel understanding is therefore interesting not only per se but also in the design of better materials for the next-generation Li^+^- and Na^+^-ion batteries.

## Supporting Information Available

XRD, additional XAS spectra, GITT curves, additional electrochemical characterization, conductivity measurements, and phase diagrams (PDF)
