# Supplementary material for: Lithiation Mechanism in High-Entropy Oxides as Anode Materials for Li-Ion Batteries: An Operando XAS Study
Source: ACS Appl Mater Interfaces. 2020 Oct 30;12(45):50344–54. doi: 10.1021/acsami.0c13161 (PMC8016163; doi:10.1021/acsami.0c13161)
Supplement: Supplementary file 1 — am0c13161_si_001.pdf [file am0c13161_si_001.pdf]

Supporting Information

Lithiation mechanism in high entropy oxides as anode materials for Li-ion batteries: an operando XAS study

P. Ghigna[a], L. Airoldi[a], M. Fracchia[a], D. Callegari[a], U. Anselmi-Tamburini[a], P. D'Angelo[b], N. Pianta[c], R. Ruffo[c], G. Cibin[d], Danilo Oliveira de Souza[e], E. Quartarone[a,*]

a. Dept. of Chemistry, University of Pavia, Via Taramelli 16, 27100 Pavia Italy

b. Dept. of Chemistry, University of Rome La Sapienza, P.le A. Moro 5, 00185, Rome Italy

c. Dept. of Materials Science, Via Cozzi 55, 20156 Milano, Italy

d. Diamond Light Source Ltd., Harwell Science and Innovation Campus, OX11 0DE Didcot U.K.

e. Elettra-Sincrotrone Trieste, Strada Statale 14 - km 163,5
34149 Basovizza, Trieste Italy

*Corresponding author: Eliana.quartarone@unipv.it

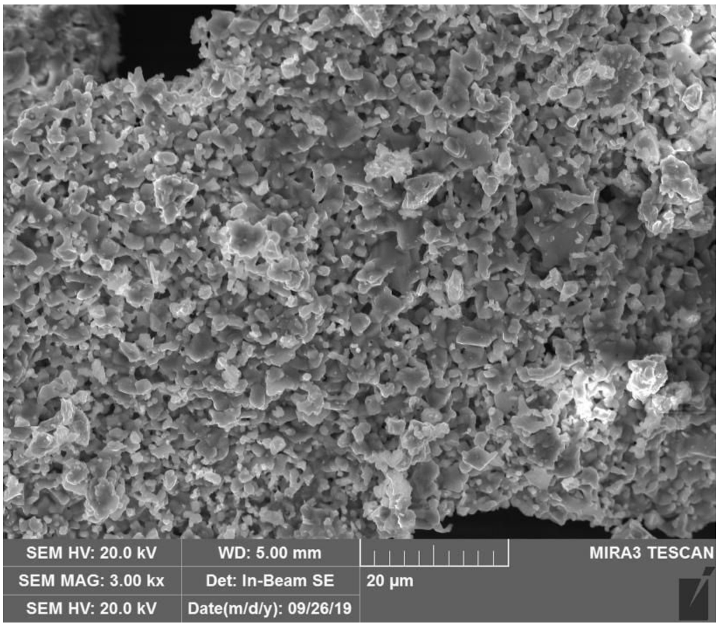

Figure S1a: SEM image of the pristine TM-HEO sample prepared via solid state reaction.

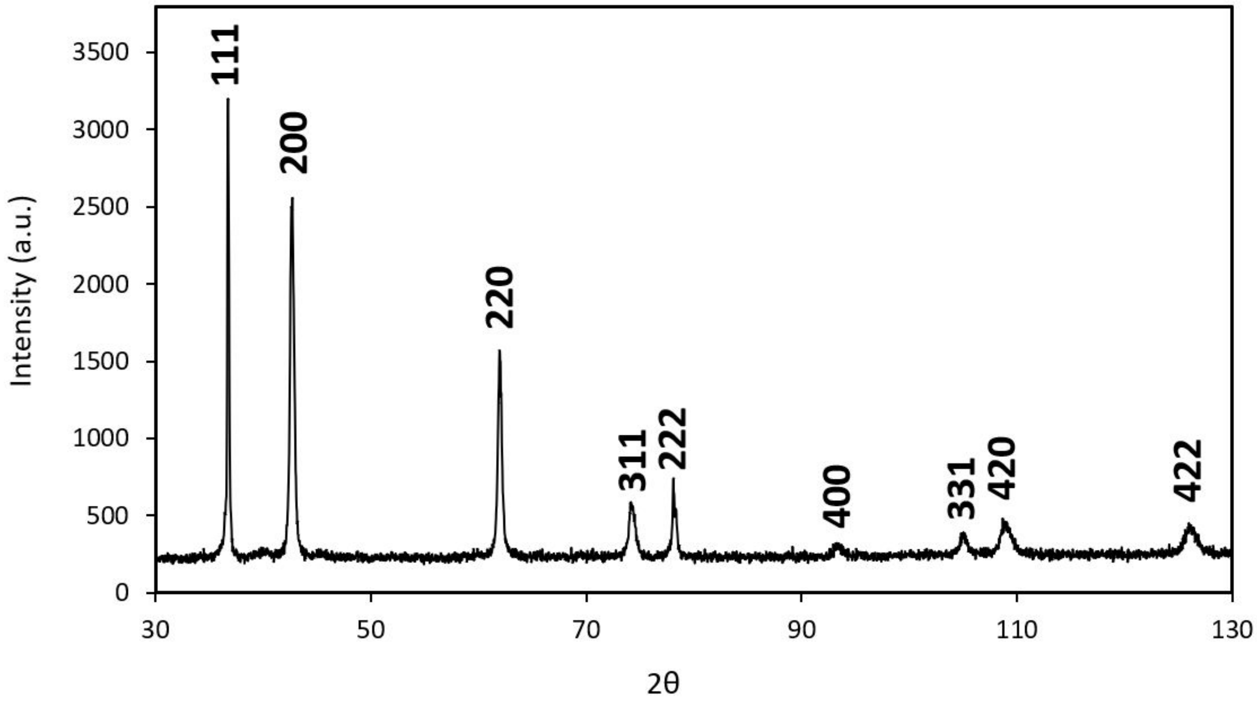

Figure S1b: XRD pattern of the pristine TM-HEO synthetized via solid state reaction.

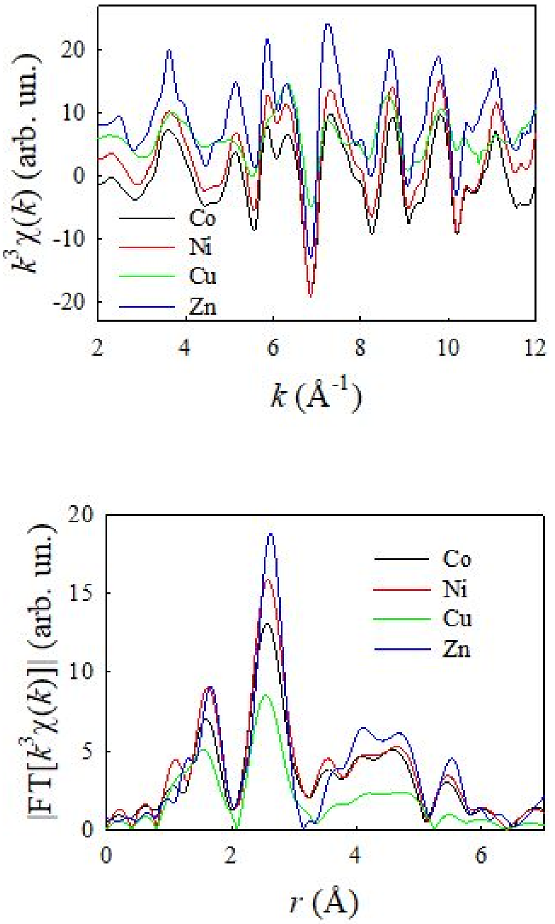

Figure S1c: EXAFS spectra at the Co, Ni, Cu and Zn K-edges of the pristine TM-HEO sample prepared via solid state reaction (upper panel); in the lower panel the corresponding Fourier Transforms are shown. For the sake of better clarity, the EXAFS spectra have been shifted along the *y* axis.

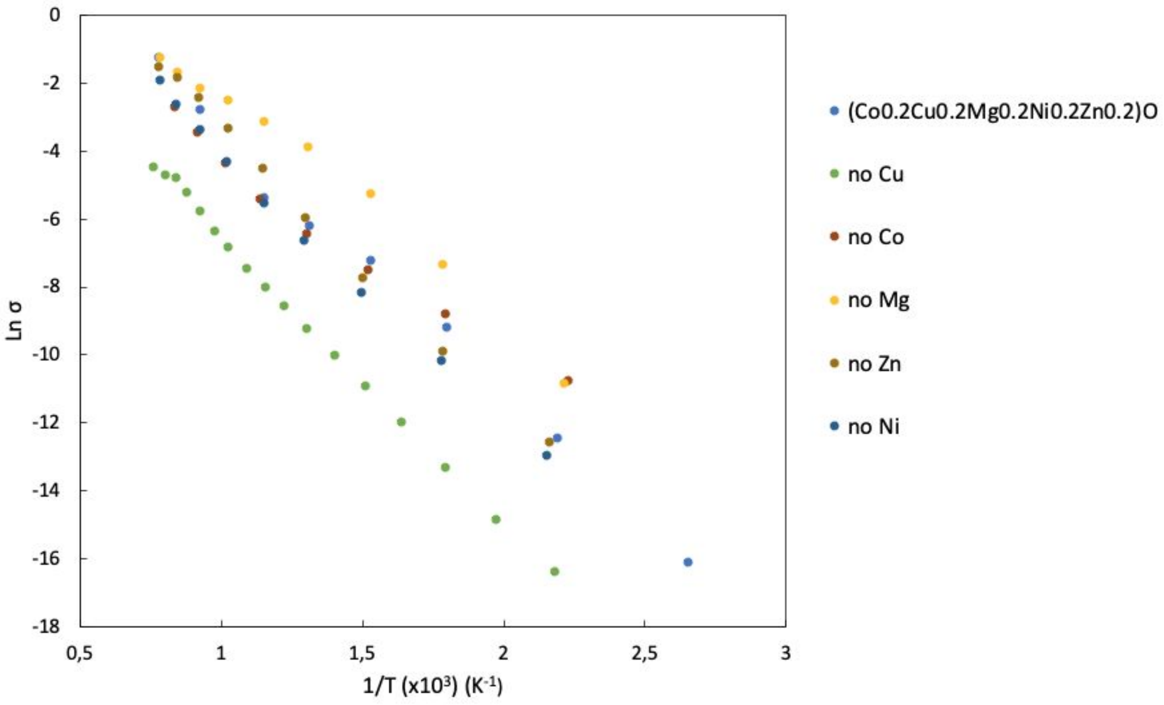

Figure S2: Conductivity behavior vs *1/T* for TM-HEO and the 4-cations based systems. The measurement uncertainty is below 5%.

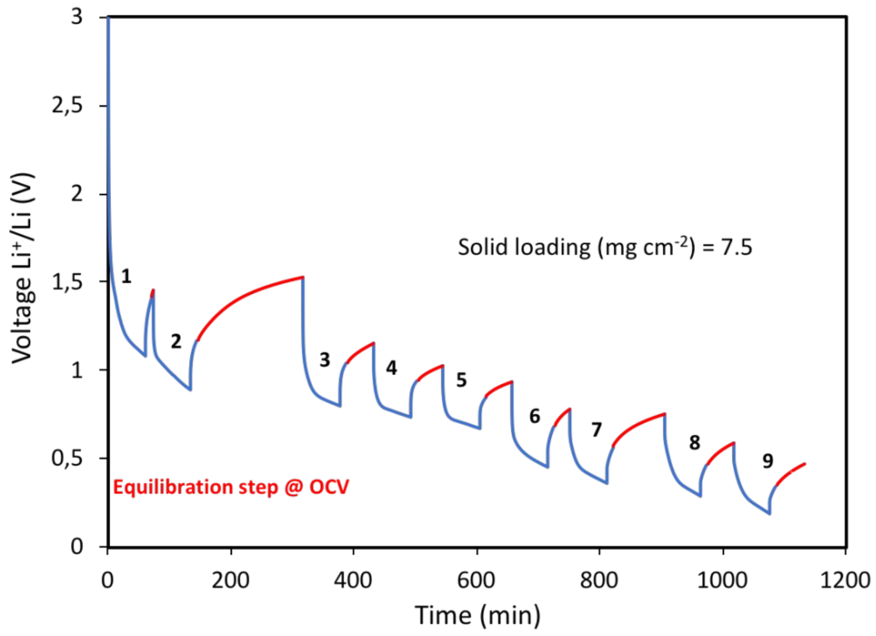

Figure S3: GITT plot during the operando XAS experiment (see methods section for details).

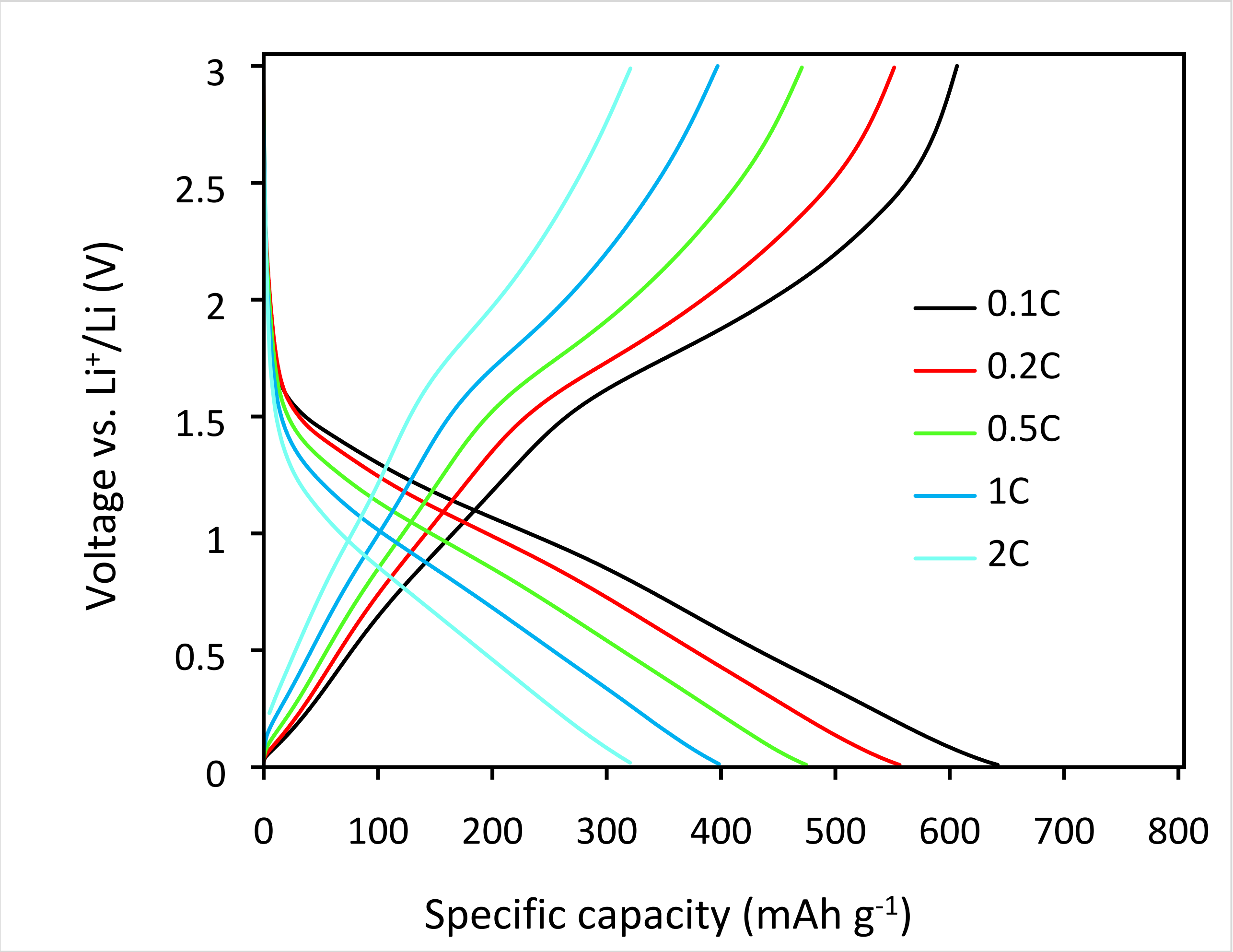

Figure S4a: Voltage profile, the lines represent the 5th cycle for each C_{rate} considered.

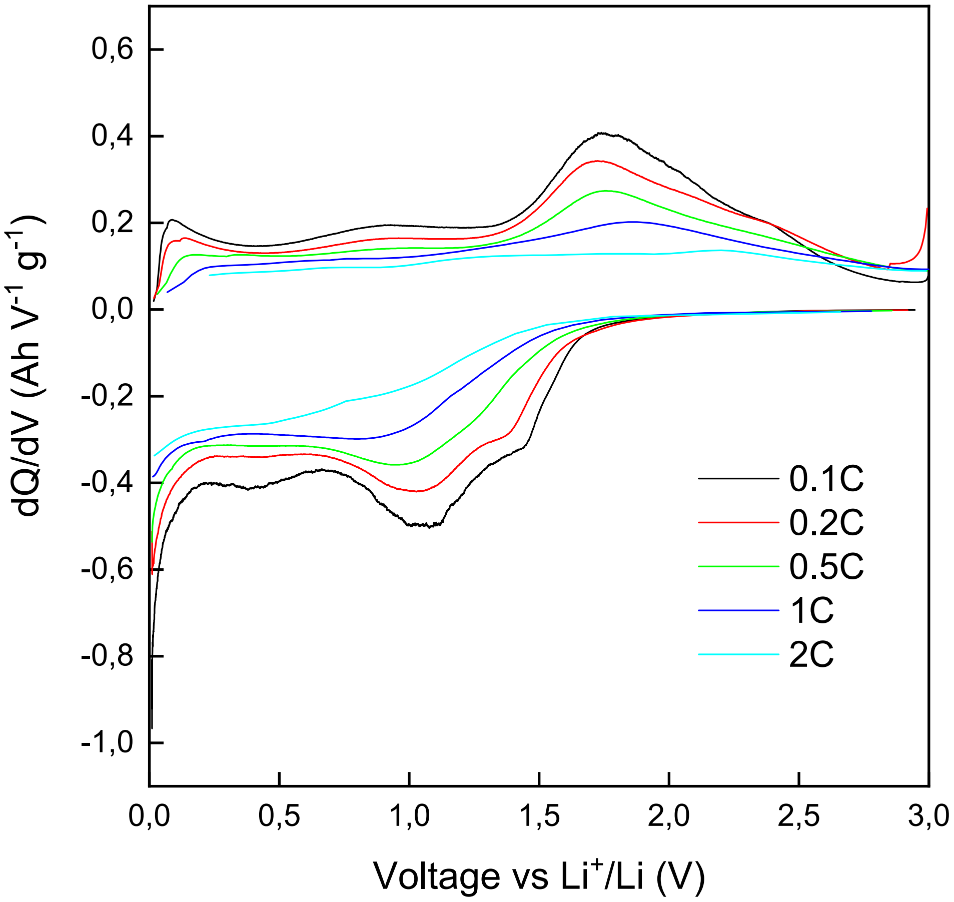

Figure S4b: Differential capacity of the same cycles of figure S5a.

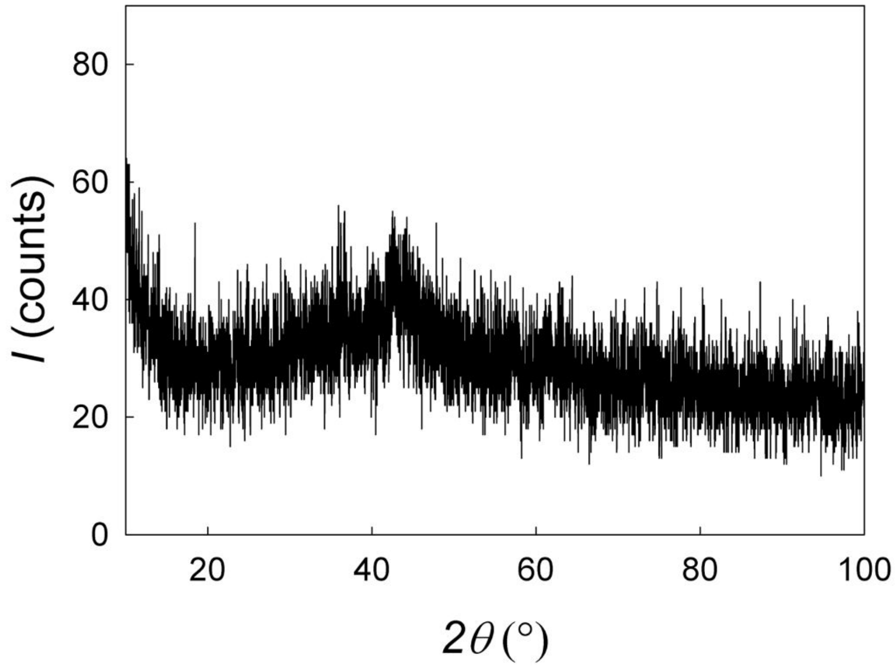

Figure S5: Post-mortem XRD pattern on the TM-HEO based anode after galvanostatic cycling. The diffraction peak around 42° corresponds to the more intense peak of FCC metals and NaCl structure.

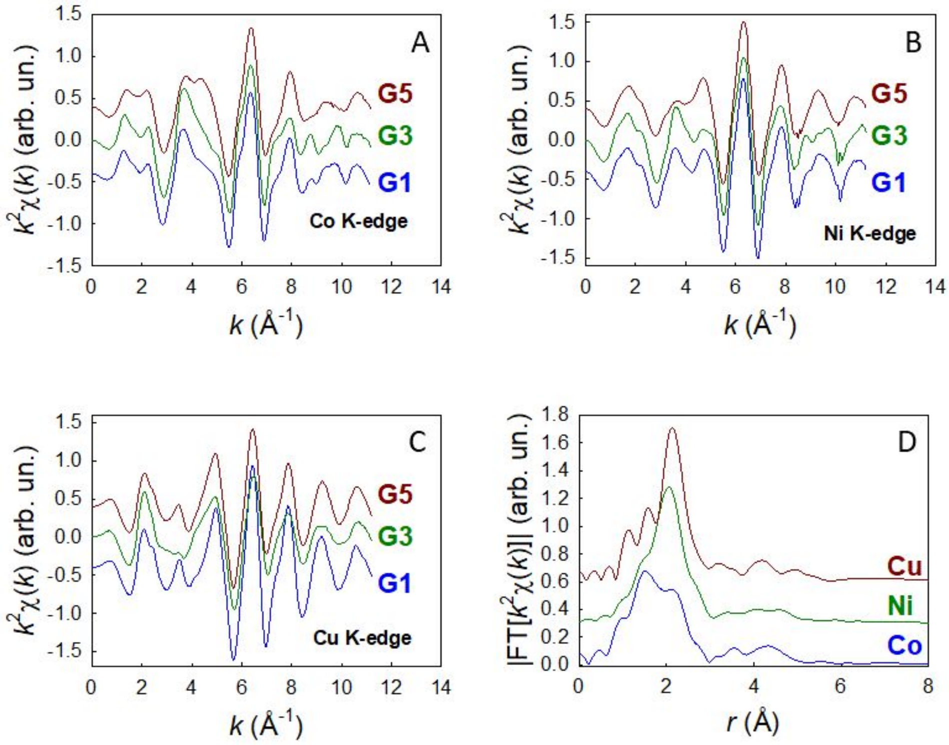

Figure S6: Co K-edge, Ni K-edge and Cu K-edge EXAFS for the G1, G3 and G5 samples (A, B and C, respectively). In D the Fourier Transforms of the EXAFS spectra of the G5 sample at all the three edges are compared. Both the EXAFS and FTs were shifted along the *y* axis for better clarity. The EXAFS spectra at the three edges show impressive similarity in all cases for $k > 4$ Å^{-1}, which is dominated by the scattering by heavy elements. This is reflected by the large peak in all the FTs at *ca.* 2 Å, which is typical of metallic phases. All these evidences point towards the fact that a large fraction of Co, Ni and Cu is in the metallic state.

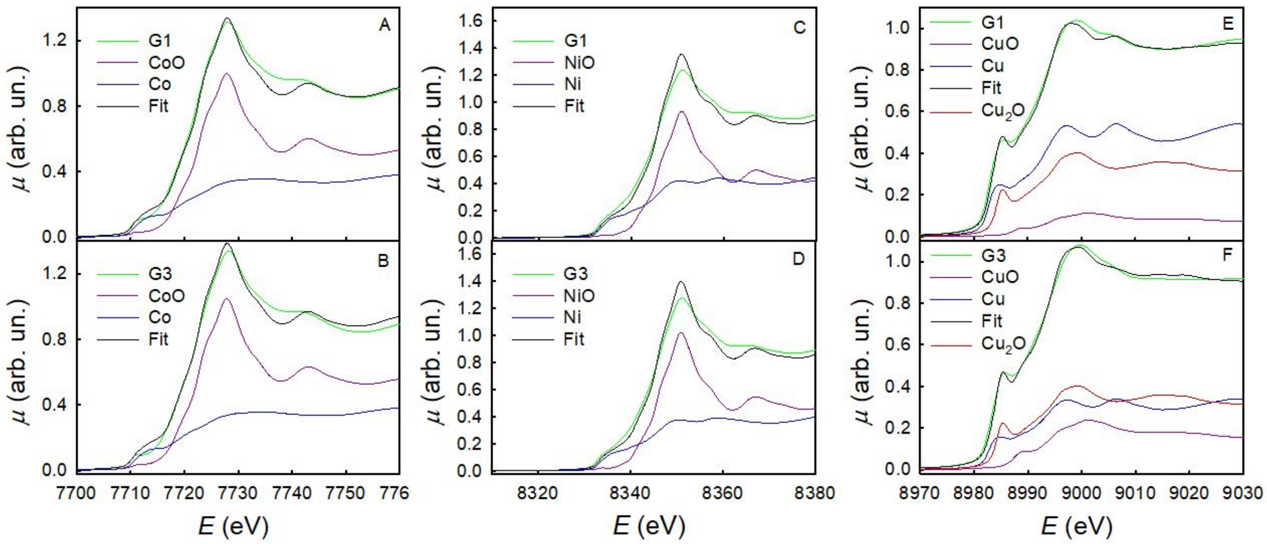

Figure S7: A, C and E show the fits of the spectra of the G1 sample at the Co K, Ni K and Cu K-edges, respectively, with linear combinations of the spectra of the reference compounds, that are also shown in the figure weighted by the coefficients of the linear combinations. B, D and F show the analogous fits for sample G3.

	**Co K**	**Ni K**	**Cu K**
Sample G1			
Co	0.38	-	-
CoO	0.62	-	-
Ni	-	0.41	-
NiO	-	0.59	-
Cu	-	-	0.54
CuO	-	-	0.09
Cu_2O	-	-	0.36
Sample G3			
Co	0.35	-	-
CoO	0.65	-	-
Ni	-	0.46	-
NiO	-	0.54	-
Cu	-	-	0.34
CuO	-	-	0.19
Cu_2O	-	-	0.47

Table S1. Linear combination fitting results of the Co, Ni and Cu K-edges XANES spectra of sample G1 and G3, expressed as mole fractions. The fit quality is expressed by the R index: for G1 is equal to 0.0059, 0.0050, 0.0011 for the Co, Ni and Cu K-edge spectra, respectively; for G3 is equal to 0.0075, 0.0059, 0.0011 for the Co, Ni and Cu K-edge spectra, respectively.

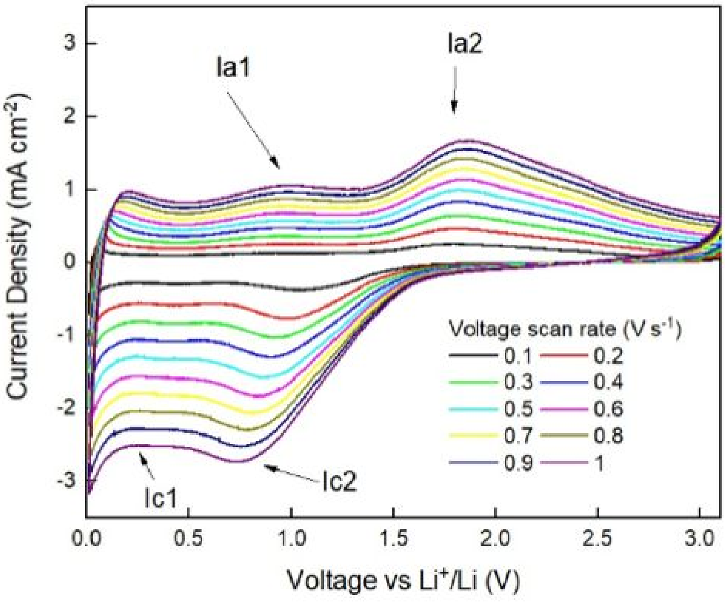

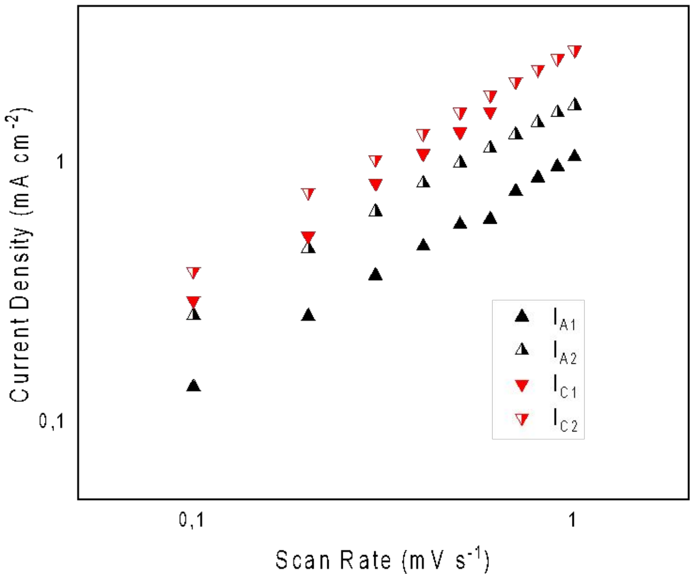

Evento	Slope
I_{A1}	0,8809
I_{A2}	0,81228
I_{C1}	0,94874
I_{C2}	0,83799

Figure S8: Cyclic voltammetry plots at different scan rate for HEO-based anode (up) and Peak Current Density, I_p, power law and corresponding b parameters (down).

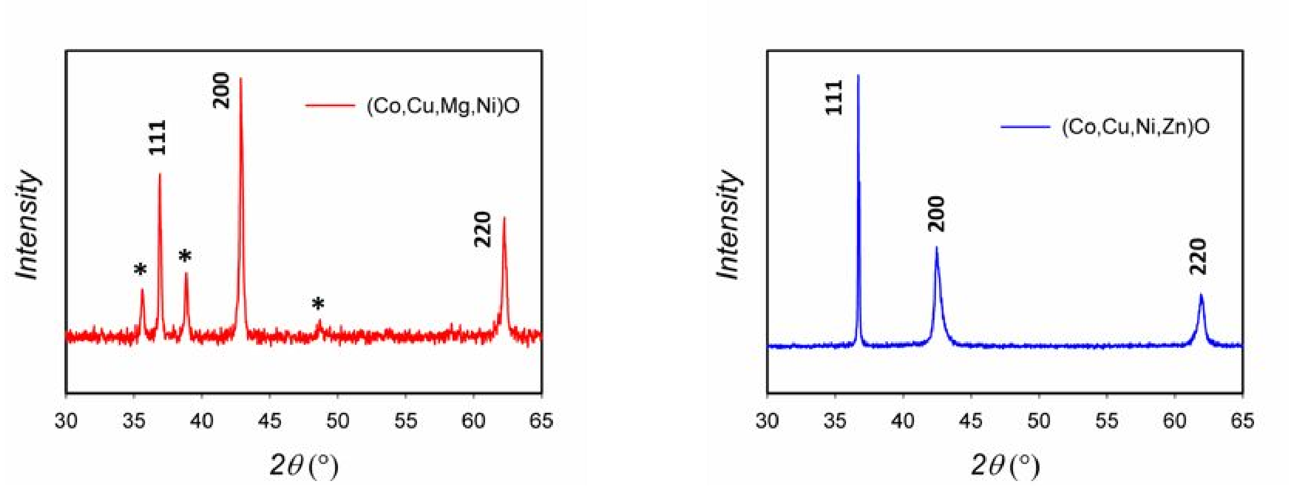

(a) (b)

Figure S9: XRD pattern of the pristine (CoCuMgNi)O (part a) and (CoCuNiZn)O (part b). The star * in plot (a) indicates the CuO signal.

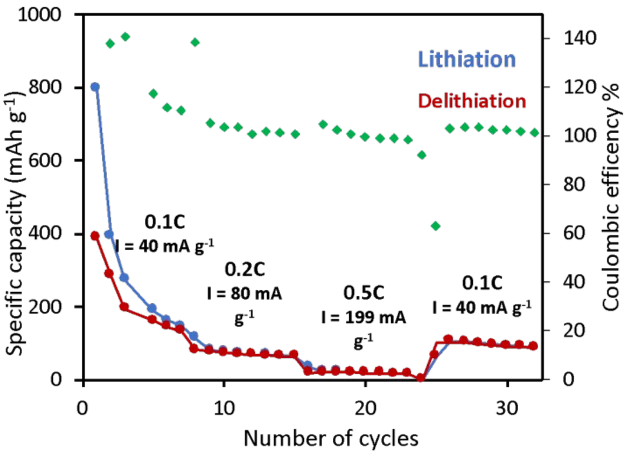

(a)

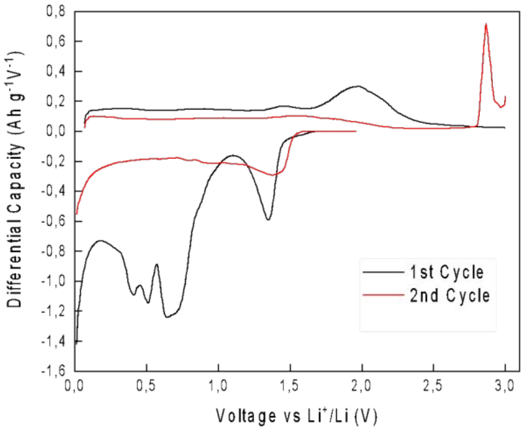

(b)

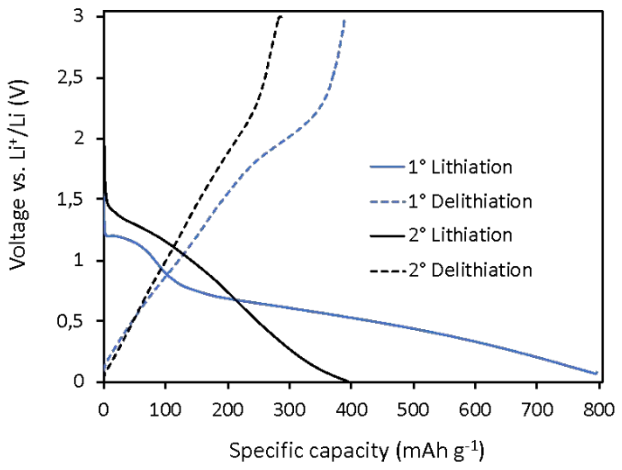

(c)

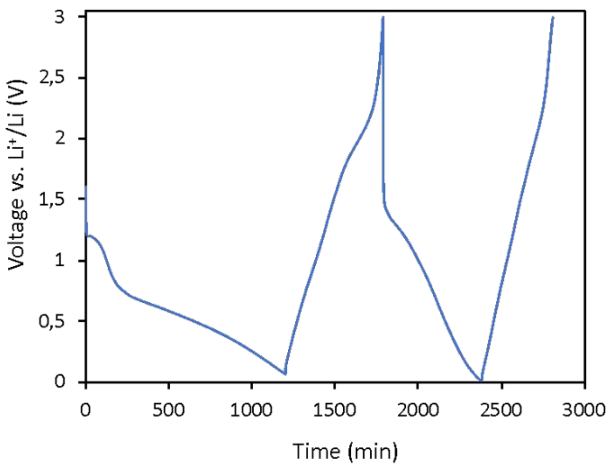

(d)

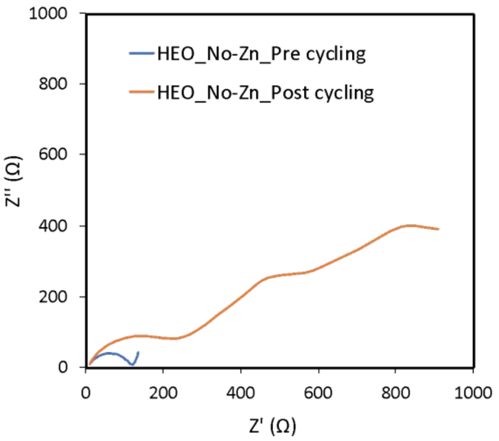

(e)

Figure S10: Galvanostatic cycling performance (a-d) and EIS Nyquist plots (e) of (Co, Cu, Mg,Ni)O-based anode.

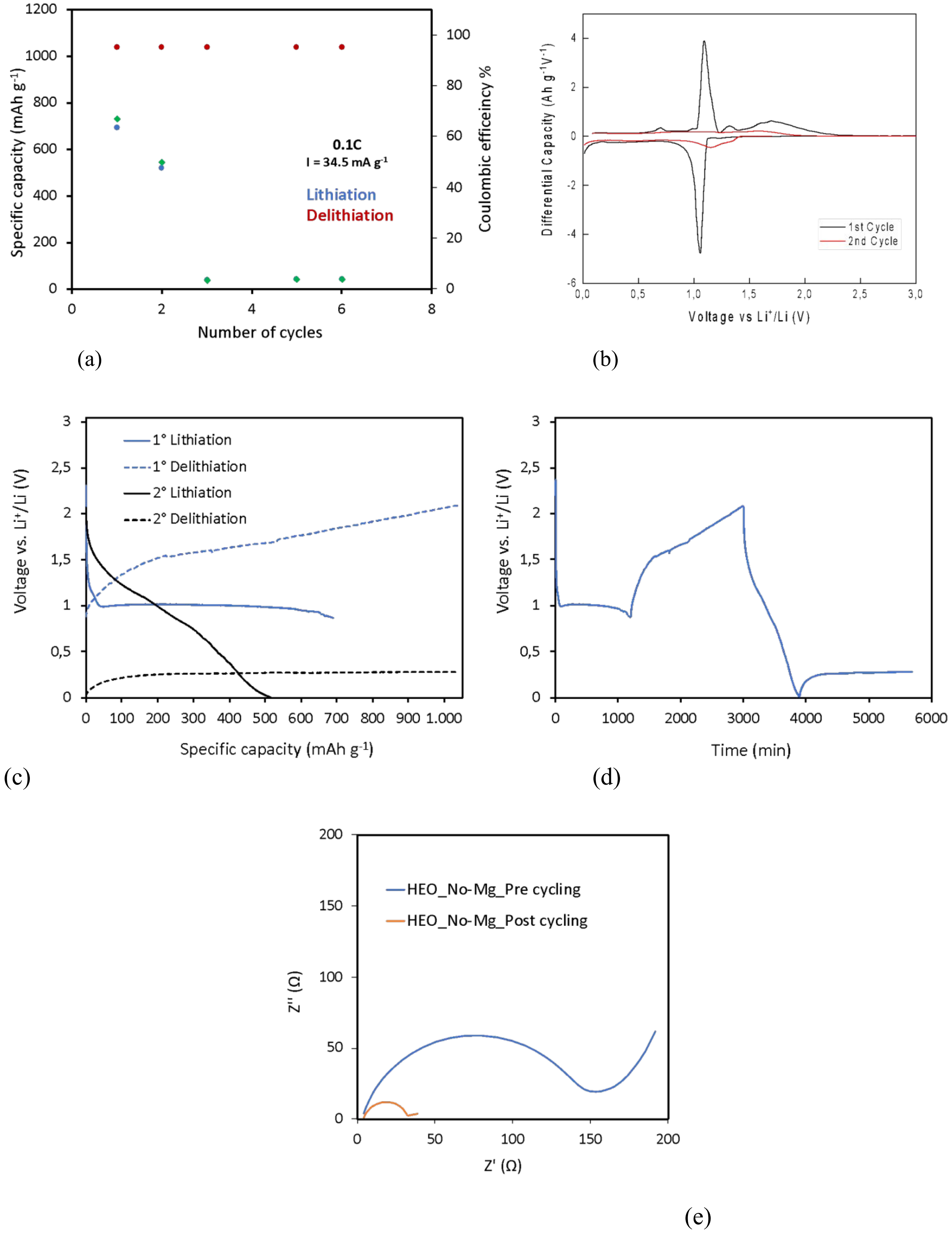

Figure S11: Galvanostatic cycling performance (a-d) and EIS Nyquist plots (e) of (Co, Cu,Ni, Zn)O-based anode.

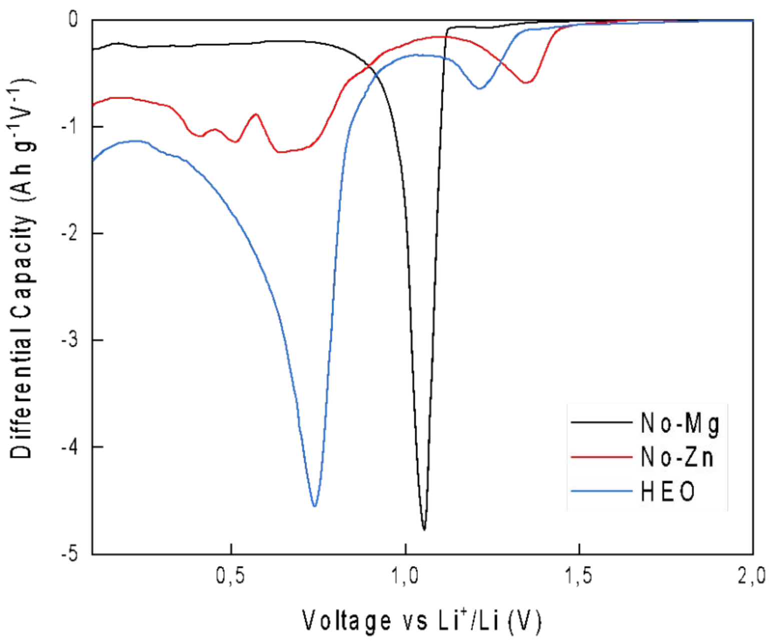

Figure S12: Comparison of the Differential Capacity determined TM-HEO-, (Co, Cu, Mg,Ni)O- and (Co, Cu, Ni, Zn)O- based anodes.

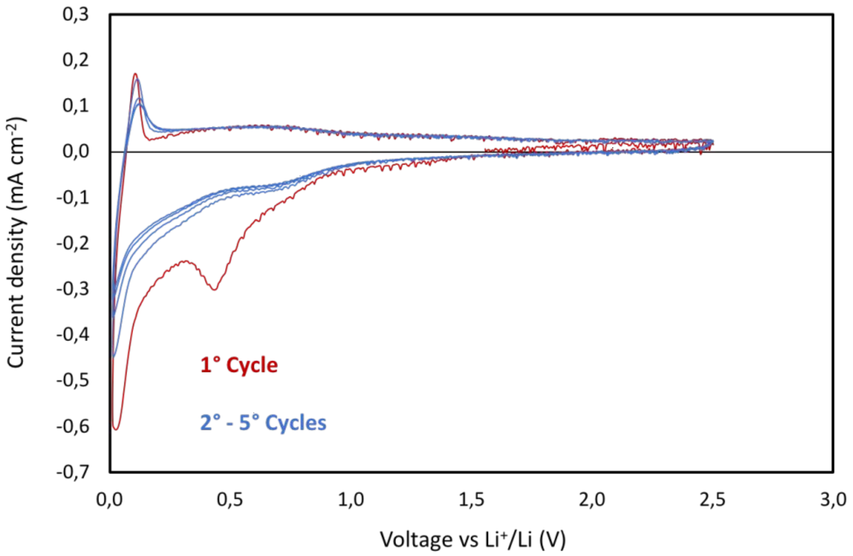

Figure S13a: Cyclic voltammograms of TM-HEO-based anodes in a Na-ion cell. Na is the counter electrode and the reference. Scan rate: 0.2 mV s^{-1}. Electrolyte: $NaPF_6$ 1.0M in EC/DEC (1/1 v/v)

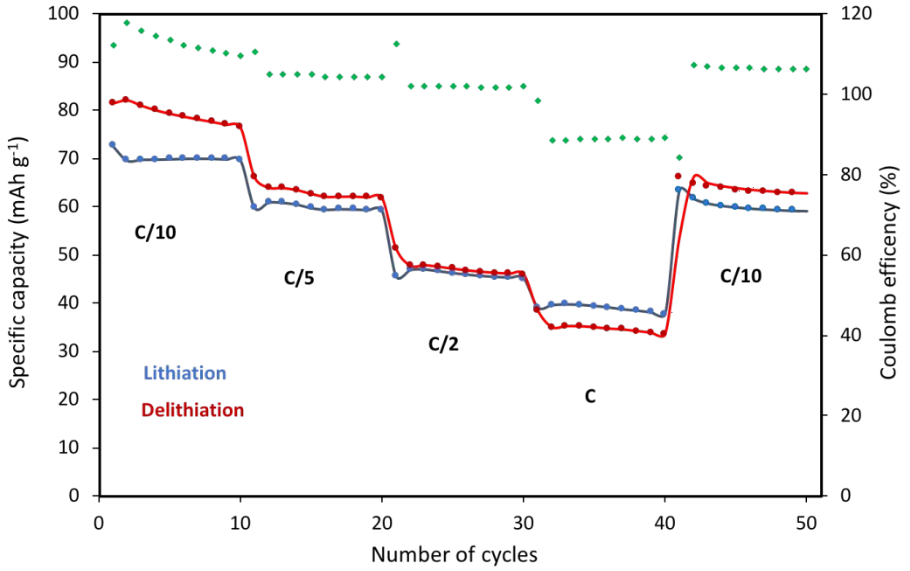

Figure S13b: Galvanostatic cycling at different C rate of the cell: TM-HEO/electrolyte/Na. Electrolyte: $NaPF_6$ 1.0M in EC/DEC (1/1 v/v).

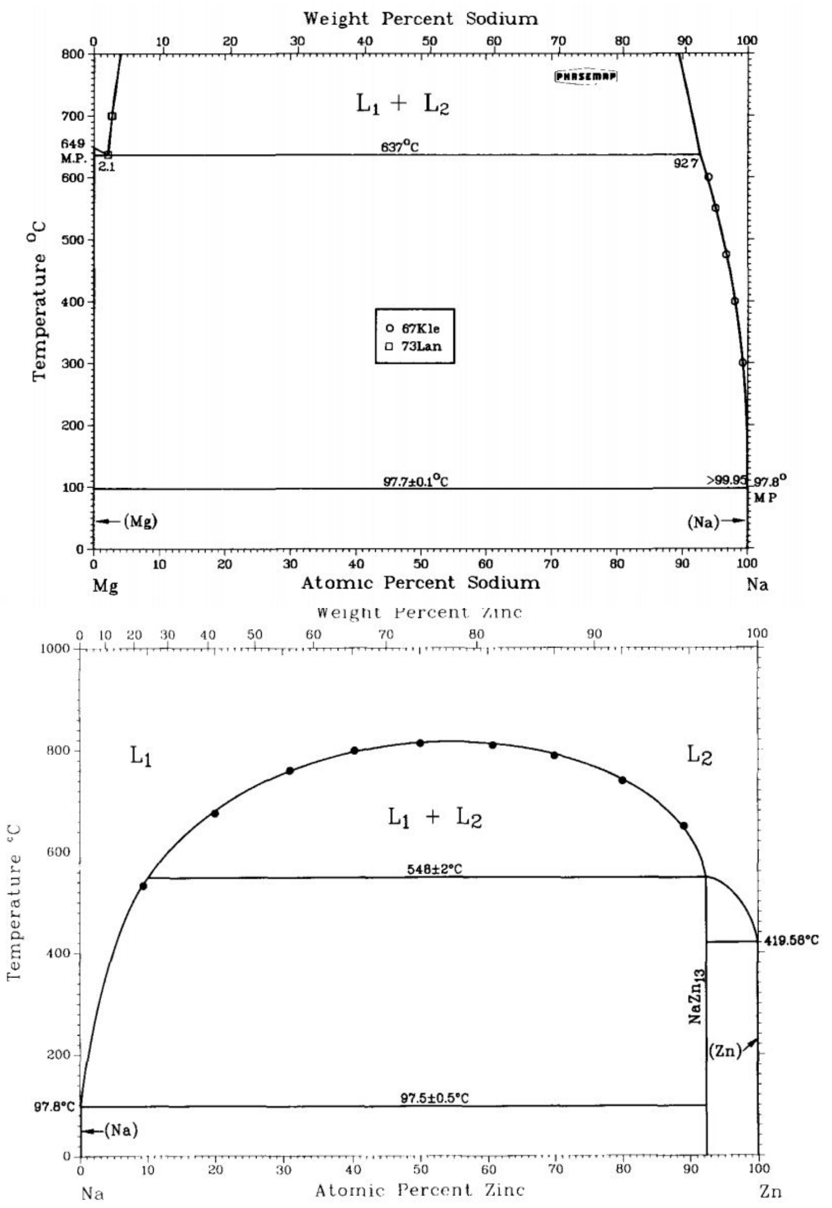

Figure S14: Phase diagram of the Mg/Na and Na/Zn binary system. Taken from refs. 41 and 42.

Appendix A

In order to justify the high reversible capacity delivered by TM-HEO anode after the first lithiation cycle and considering that the TMs do not undergo any further reactions, it is reasonable to speculate that the such capacity could derive from alloying/dealloying phenomena involving ZnO and MgO, which occur through the following two-step process (M=Zn, Mg):

1) $MO + 2Li^{+} + 2e^{-} \rightarrow M + Li_2O$
2) $M + xLi^{+} + xe^{-} \rightarrow LixM$

In case of the ZnO anode, it well-known that the reaction will lead to the formation of LiZn alloy. Taking into account its theoretical capacity and the molar content in the HEO, ZnO will contribute to the total delivered capacity (average value of 615 mA h g^{-1} @C/10) for a value of 197 mA h g^{-1}.
The remaining capacity (418 mA h g^{-1}) could be ascribed to the Li_xMg formation. What happens for MgO is more complicated, because x is not predictable; however, it can be estimated by the inverse formula of the specific capacity:

$$n = \frac{3.6C_{s,\ rev}Mw_{HEO}}{F} \sim 1.0$$

Considering that for each mole of HEO, 0.2 moles of Mg are produced, the calculated stoichiometry of lithium per unit of magnesium should be:

$$x \sim \frac{1.0}{0.2} = 5$$

Resulting in the Li_5Mg alloy with an Li atomic percentage of about 80%. This hypothesis does not violate the phase diagram, reported in Fig.S12.

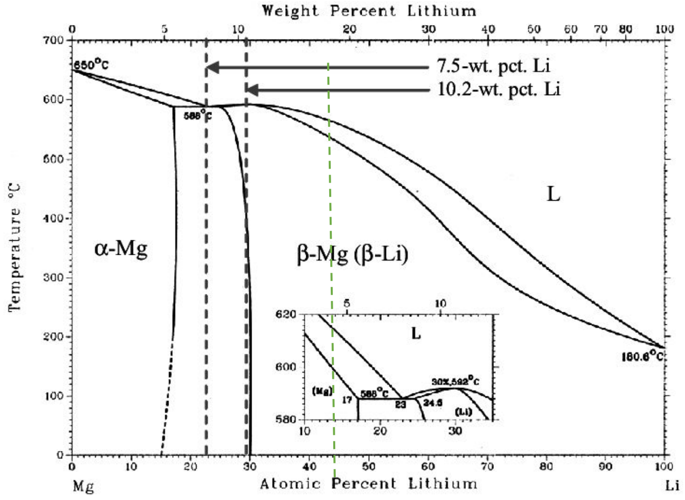

Figure S15: Phase diagram of Mg/Li binary system. Taken from refs. 38-39.
